# Advances in electrocardiogram signal analytics for person identification using fine-grained fusion-driven deep representation learning under arrhythmic conditions

**DOI:** 10.3389/fdgth.2026.1841911

**Published:** 2026-08-18

**Authors:** Majed Balkheer, Mahmoud Ragab, Reda Salama, Ashis Kumer Biswas

**Affiliations:** 1 Department of Computer Science and Engineering, University of Colorado Denver, Denver, CO, United States; 2 Department of Information Technology, Faculty of Computing and Information Technology-Rabigh, King Abdulaziz University, Jeddah, Saudi Arabia; 3 Department of Information Technology, Faculty of Computing and Information Technology, King Abdulaziz University, Jeddah, Saudi Arabia

**Keywords:** deep learning, electrocardiogram signal, person identification, R-peak detection, short-time Fourier transform, spectrograms

## Abstract

**Introduction:**

Electrocardiogram (ECG) signals measure the minute electrical signals produced throughout the cardiac cycle, whereas biometric signals are vital indicators of human activity. Recent studies highlight ECG as a very significant tool for clinical analysis and as a novel biometric modality. Because of differences in physical features like height, heart size, muscle mass, weight, and fat mass between individuals, each person exhibits unique characteristic signals. ECG-based identification has numerous applications, including unlocking electronic doors, banking transactions through smartphones, and facilitating website login. The usage of image-based representation allows recognized computer vision structures to exploit both rhythm-related and morphological cues, enhancing discrimination across individuals. In recent times, deep learning and machine learning have been progressively applied to ECG signals for a wide range of tasks such as person identification and arrhythmia detection. Deep learning approaches like Long Short-Term Memory (LSTM), Convolutional Neural Networks (CNNs), and Transformer based systems can learn hierarchical representations directly from raw or slightly processed ECG waveforms, capturing both temporal dynamics and morphological details of heartbeats.

**Methods:**

This paper proposes FDDR-ID (FusionDriven Deep Representation Learning for ECG-Based Person Identification), a multitask deep learning framework designed to achieve robust identification performance under arrhythmic conditions. The proposed model initially preprocesses the ECG signals through three sub-processes: non-local means (NLM) filtering-based denoising, R-peak detection, and segmentation. To exploit the supremacy of deep learning approaches, every pre-processed ECG segment is converted into a two-dimensional representation using the shorttime Fourier transform to generate spectrograms. For feature extraction, a fusion of three deep learning models is used: SqueezeNet, NASNet, and EfficientNet. Moreover, a Siamese LSTM Autoencoder is utilized for the classification process, which includes three output branches: one each for person identification, age prediction, and gender classification. The model is trained with adaptive learning using the Nadam optimizer, ensuring faster convergence and improved generalization.

**Results and Discussion:**

Performance validation is conducted on the proposed FDDR-ID model utilizing the ECG-ID Database and the MITBIH Arrhythmia Database. Extensive comparative analyses demonstrate that the presented method outperforms other state-of-the-art approaches. The proposed FDDR-ID method also shows important potential for real-world deployment in applications like smart authentication systems, wearable healthcare devices, remote patient monitoring, and secure biometric access platforms.

## 1 Introduction

Information privacy and security are critical concerns for organizations that are required to protect their networks, systems, websites, and other services, along with their information (1). The biometric recognition process is frequently proposed as an efficient tool for logical and physical access control, as well as observation, to ensure information security. Biometrics is the dimension of a person's behavioral or physiological characteristics (2). Biometric authentication has also been employed to reduce the risk of cyberattacks in hospitals and other healthcare services. Because of the extensive usage of smart devices such as smartwatches and smartphones, biometric detection has increased in popularity (3). Therefore, conventional biometric modes—such as face, voice, handwriting, iris, and fingerprint—are vulnerable, as they may be simply replicated and utilized fraudulently (4). For instance, fingerprints may be reproduced using latex, voices can be imitated easily, faces are susceptible to artificial masks, and irises can be forged using contact lenses with copied iris characteristics (5). Moreover, a few of these traits may be lost following serious trauma, injuries, or altered by surgical procedures. In addition, these biometric methods are not always beneficial in various applications. Furthermore, other evolving biometric modes are being examined to solve these restrictions while also complementing conventional biometrics (6). The electrocardiogram (ECG) signal is one such biometric tool.

ECG signals have long been utilized in medical diagnosis. In recent times, it has been recommended that ECG signals be employed as a biometric modality for individual identity detection (7). The ECG signal, otherwise called a heartprint, is an evolving biometric mode used to enhance security. As ECG signals can only be obtained from a living individual, they offer inherent liveness detection (8). As the heart is limited to the body, ECG signals are too complex to alter or fabricate, unlike fingerprints, irises, or facial biometrics. Recently, research has been conducted on ECG sensor-equipped jackets to monitor human stress levels and automatically identify the subject wearing the jacket, ensuring that health information from that jacket aligns with the correct person without a manual login process (9). High-quality ECG indications can also be taken from the fingers. All this creates a more acceptable and secure biometric recognition. There are many existing ECG-driven biometric methods with outstanding performance on standard ECG signals (10). These approaches evolved their own deep learning (DL)-driven technique and utilized the transfer learning (TL) approach with pretrained convolutional neural networks (CNNs) (11). DL has become the most broadly utilized technique for the ECG biometric method. Diverse DL structures have been accepted in ECG biometric models. DL-driven techniques normally outperform conventional hand-engineered as well as machine-learning (ML)-driven methods (12). On arrhythmic ECG indications, the same model performs less well than the usual ECG sign-driven detection model. CNN is the main DL method, and it has garnered significant attention by utilizing its power in automating learning the inherent patterns from the information, which may both reduce time-consuming physical feature engineering and observe hidden inherent patterns more efficiently (13). Cardiovascular disease (CVD) is an important cause of mortality worldwide, emphasizing the significance of early analysis and continuous monitoring. Traditional diagnostic algorithms frequently need bulky hospital equipment and time-consuming processes. Current advancements in biosensing technologies and wearable devices enable rapid, accurate, and continuous monitoring of cardiac signals, assistant enhanced disease management and personalized healthcare (14).

### 1.1 Paper contribution

This article presents a fusion-driven deep representation learning for ECG-based person identification approach (FDDR-ID). The following are the major contributions of the paper:

- The proposed FDDR-ID method aims to achieve robust person identification from ECG signals under arrhythmic conditions, enabling robust identity discrimination despite significant morphological variations in abnormal cardiac rhythms.
- The ECG preprocessing pipeline introduces non-local means (NLM) filtering-based denoising, R-peak detection, and segmentation, which yield clean and stable input segments.
- Two-dimensional spectral representation converts every pre-processed ECG segment into spectrograms utilizing Short-Time Fourier Transform (STFT).
- In terms of feature extraction, we have implemented three DL techniques, SqueezeNet, NASNet, and EfficientNet, to obtain rich and diverse representations, to capture lightweight local descriptors, multi-scale spatial representations, and high-level semantic features, thereby improving discriminative feature learning.
- A fine-grained feature fusion mechanism is developed to selectively combine heterogeneous deep representations extracted from multiple CNN architectures, generating a unified embedding that preserves both local morphological characteristics and global temporal patterns of ECG signals.
- Siamese with LSTM autoencoder is used for multi-task outputs: utilizing a Siamese with LSTM Autoencoder architecture with three output branches for person identification, age prediction, and gender classification enables joint learning and shared representations.
- The adaptive training strategy employs the Nadam optimizer and adaptive learning method to improve convergence and generalization.
- Comprehensive evaluation assesses the proposed FDDR-ID model using ECG-ID and MIT-BIH Arrhythmia Databases, and this shows evidence of enhanced performance compared to recent state-of-the-art methods.

The novelty of this work lies in the development of an integrated ECG biometric model that incorporates complementary feature extraction, explainable feature fusion, similarity learning, and temporal representation learning into an end-to-end framework. The unified model allows for concurrent biometric recognition and demographic prediction while enhancing model interpretability via Grad-CAM-based feature fusion. The use of Grad-CAM visualization shows us that every network corresponds to distinct spectrogram regions: SqueezeNet corresponds to localized high-frequency bursts, NASNet to mid-frequency temporal patterns, and EfficientNet to smoother, distributed energy regions. The combination of complementary features acts as an implicit feature-level ensemble, offering the downstream classifier a richer representation compared to that of a single CNN.

## 2 Related works

In this part, an overview of existing studies on person identification using ECG signals is discussed. Al-Jibreen et al. (1) presented a human classification approach that considers ECG signals annotated with health conditions where every individual's beats overlap between irregular arrhythmia classes. The overlap between the normal class and other arrhythmia classes enables isolation of normal beats in the training set from the Arrhythmic beats in the testing set. Moreover, the article examined the impact of arrhythmic heartbeats on biometric detection. An efficient lightweight CNN driven by depth-wise separable convolution (DWSC) is presented to improve the efficiency of individual classification for various common arrhythmia types. Aleidan et al. (6) evolved cardiac biometrics for person classification employing a DL method. Cardiac biometric methods are based on cardiac signals that are taken utilizing phonocardiogram (PCG), ECG, and photoplethysmogram (PPG). The research uses the ECG as biometric security since ECG signals are preferred for precise, safe, and consistent biometric-driven person classification models, setting them apart from PCG as well as PPG methods. Hazratifard et al. (15) presented an idea employing ECG signals that are simply available in a digital medical system for verification across an Ensemble Siamese Network (ESN) that may manage small variations in ECG signals. Enhancing pre-processing for the feature extractor in the technique may result in improved outcomes. Continuous authentication that depends on contextual data provides more benefits than conventional authentication. Recent authentication techniques that are based on ML have their limitations, like the complexity in enrolling novel users to the model or system.

Hamza and Ayed (16) proposed an effective human classification technique that depends on ECG signals. This method is realized in various phases, like classification, segmentation, pre-processing, recognition of R peaks, and feature extraction. After the pre-processing stage and the recognition of the R-peaks stage, the segmentation phase is performed. The method utilized dual instances of the segmentation, like fragments with two R-peaks as well as fragments with a single R-peak. Therefore, the stage of feature extraction involves extracting the non-fiducial characteristics. Lee and Kwak (11) introduced a human classification model that depends on a CNN as well as an ensemble of LSTMs that utilizes ECGs. An ECG uses inner biometric data, the heart rate signals employing microcurrents and thus involving noise throughout measurements. This noise is removed using filters in a pre-processing phase, and the signals are separated into cycles with respect to R-peaks for feature extraction. Byeon and Kwak (17) introduced an ensemble DNN for individual classification by analyzing and comparing the ECG detection efficiency under several states. This method contains three streams that combine two well-known pre-trained techniques. Every network obtains the time-frequency representation of ECG signals as input, as well as a stream that reprocesses a similar network structure under various learning states with or without data augmentation. This phenomenon poses a risk for individual classification employing ECG signals acquired on different days.

In a study by Gupta and Avasthi (18), an individual classification method that depends on ECG is presented, where the statistical measurements as well as distinguished ECGs are utilized as characteristic sets, and for the identification of ECGs, LSTM is considered. This approach is more precise than advanced approaches that depend on outcomes from a type of abnormal and normal pathological ECG information. Biran and Jeremic (19) presented an innovative method for human classification employing ECGs logged on different days. The major aim of the work is to extract the most important wavelet discriminatory factors of the ECG signs by using the ECG spectral alteration for person classification employing a multi-level filter method. This study (20) offers a novel Zeolite/PDMS-based dry electrode for continuous ECG monitoring as an alternative to conventional wet electrodes. The proposed method uses the iono-electric properties of Zeolite 4A and Zeolite 13X combined with PDMS to improve optically discreet and skin-friendly electrodes. Extensive electrical and bio-electrical assessments were carried out using dissimilar electrode configurations depending on filler diameter, concentration, and width.

Begum et al. (21) developed an architecture for integration of advanced signal processing and neural architectures, which makes it suited for clinical applications in resource-constrained settings. Umamaheswari et al. (22) implemented an Optimal Artificial Neural Network (OANN) model, which is used in diagnosing heart disease (HD) by the utilization of large amounts of data. As an outcome of its structure on top of the popular Apache Spark framework, the algorithm is suited for making predictions both offline and online. Kannimuthu et al. (23) presented an idea that the massive amount of information generated by body area networks means machine learning is needed for health data classification. It is predicted that the implementation of the GBC as suggested results in the most favorable effects achievable. Several research works on person identification using ECG signals are presented in Table 1.

**Table 1:** Comparison analysis of related work.

Authors	Techniques	Datasets	Findings	Objectives
Al-Jibreen et al. (1)	Deep Convolutional Neural Network	MITBIH dataset.	Accuracy of 99.58%.	To project a person recognition model analyzing the health-state annotated ECG signal of every person's beats overlapping amid various arrhythmia categories.
Aleidan et al. (6)	VGG16 and Long Short-Term Memory (LSTM).	ImageNet dataset.	Accuracy of 98.7%.	To present an innovative cardiac biometric method for person recognition utilizing numerous models.
Hazratifard et al. (15)	Ensemble Siamese Network (ESN) and CNN.	ECG-ID and PTB datasets.	Accuracy of 93.6% and 96.8%.	To project a robust authentication model that can enroll novel consumers at run time, while being strong to imbalanced conditions.
Hamza and Ayed (16)	Support Vector Machines (SVM)	MIT-BIH and ECG-ID datasets.	Accuracy of 100%, 100%, 100%, and 99.01%.	To develop an effective person recognition model that depends on ECG signal and is used to diagnose various stages.
Lee and Kwak (11)	Ensemble of CNN and LSTM.	ECG dataset.	Accuracy of 95.12% and 97.67%.	To develop a personal recognition model for allowing remote investigation of heart diseases like arrhythmia or cardiac arrest.
Byeon and Kwak (17)	Ensemble of Deep Neural Networks	PTB dataset.	Improvement of 3.39%.	To investigate an ensemble model for calculating and comparing ECG signal identification outcomes under various conditions.
Gupta and Avasthi (18)	LSTM	ECG dataset.	Achieves a better outcome.	To examine person recognition models that depend on the ECG signal; here, the discriminated ECG and its statistical measures are employed.
Biran and Jeremic (19)	WMVM, MODWT, and IMODWT.	ECG ID dataset.	Accuracy of 92.29%.	To present an innovative model for human recognition using ECGs recorded on various days.
Pullano et al. (20)	Zeolite/PDMS-based dry ECG electrodes using Zeolite 4A and Zeolite 13X SNR, and ECG acquisition analysis	ECG signals collected from fabricated 24 electrode pairs under different configurations	The Zeolite 13X/PDMS electrode with 4 wt% with 42.9 dB SNR and 0.58 μS/mm conductivity,	To progress an optically unobtrusive, skin-friendly, dry ECG electrode for continuous ECG monitoring, enabling next-generation wearable cardiovascular systems
Begum et al. (21)	FusionWave-KalICA, VGG-13 SpectraNet with (THA), TCN	Clinical Parkinson's disease dataset	Achieved up to 98.68% accuracy for Parkinson's disease detection across various public EEG datasets	The proposed ParkinsonFusionNet effectively combines signal processing and deep learning techniques, providing robust feature extraction and making the framework suited for clinical applications.
Umamaheswari et al. (22)	Optimal Artificial Neural Network (OANN)	Large-scale heart disease dataset	Experimental results show that the ANN model surpassed conventional machine learning methods in respect of scalability and accuracy.	To precisely diagnose heart disease by an optimized ANN model which is capable of handling large-scale healthcare data.
Kannimuthu et al. (23)	Machine Learning-based cardiovascular risk prediction by Gradient Boosting Classifier (GBC)	Body Area Network (BAN)-generated cardiovascular health data	The optimized model attained high predictive performance and excelled conventional machine learning approaches.	To sort healthcare data and to predict binary cardiovascular disease risk constraints using machine learning methods.

## 3 Proposed system

**Figure 1:**
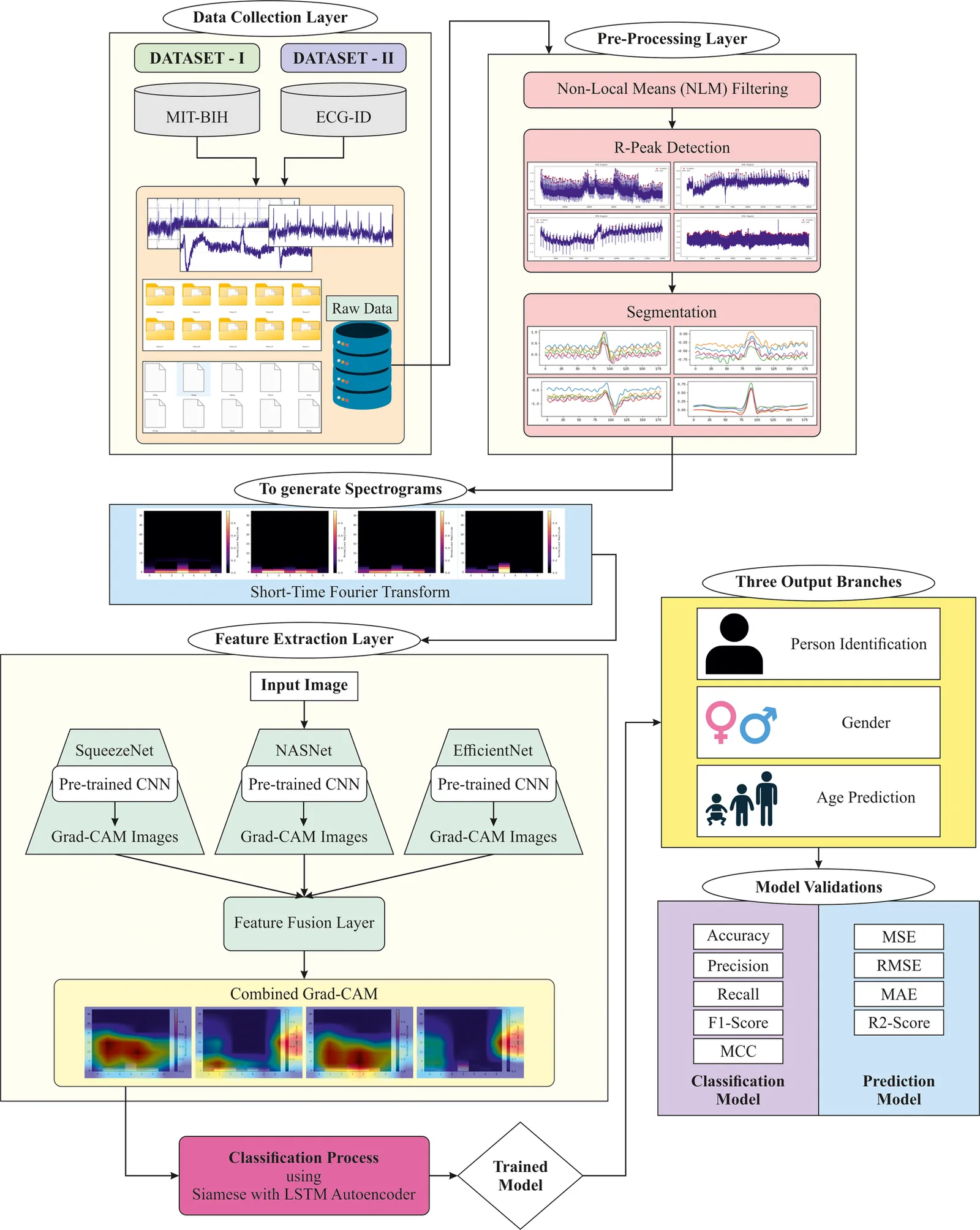
Overall workflow of the FDDR-ID system.

In this study, the proposed FDDR-ID model follows a structured approach for robust and accurate person identification using ECG signals, even under arrhythmic conditions. Figure 1 demonstrates the overall workflow of the FDDR-ID system. Initially, the preprocessing stage is carried out, where ECG signals undergo three key operations: NLM filtering-based denoising, R-peak detection, and segmentation to eliminate noise, identify key cardiac cycles, and divide the signal into meaningful segments for further analysis. After preprocessing, each ECG segment is transformed into a two-dimensional spectrogram representation using the STFT. Subsequently, in the feature extraction stage, a fusion of three DL models—SqueezeNet, NASNet, and EfficientNet—is utilized to extract fine-grained and discriminative features from the spectrograms, ensuring comprehensive representation learning. Following feature extraction, the Siamese network integrated with an LSTM Autoencoder is utilized for the classification process, which produces three outputs corresponding to person identification, age prediction, and gender classification. The model is trained using the Nadam optimizer, which supports adaptive learning and ensures faster convergence along with improved generalization capability. The proposed architecture jointly learns person identification, age prediction, and gender classification by a shared feature extraction network. Person identification is considered the primary task, whereas age prediction and gender classification are considered auxiliary tasks. The shared learning approach improves feature generalization, which improves the execution of the primary biometric identification tasks and reduces overfitting. The tasks are therefore highly related, and the proposed architecture is designed to reduce the negative transfer by joint optimization of the task-specific and shared layers.

### 3.1 ECG preprocessing

Initially, the model pre-processes the ECG signals with three sub-processes, including NLM filtering-based denoising, R-peak detection, and segmentation.

#### 3.1.1 NLM algorithm

The NLM approach is the most prevalent image denoising model in CV (24). The NLM method is used through preprocessing to remove noise while conserving the morphological characteristics of ECG signals. Unlike conventional filters that rely only on local neighboring instances, NLM detects signal self-similarity in relating patterns over the whole signal and transfers similarity-based weights. This methodology efficiently destroys noise while retaining crucial ECG mechanisms like QRS complexes, P-waves, and T-waves, thus refining signal quality for subsequent feature extraction and identification tasks. The calculation of $\mathrm{the}\;i_{th}$ instance is found by the average weight of instances with the same local neighborhood in the searching window N(i) is given in Equation 1.

$$\hat{u}[i]=\frac{1}{C(i)}\underset{\;j\in N(i)}{\sum }w(i,\;j)v[i]$$ (1)

Here, w(i,j) refers to the weight of the $j_{th}$ neighborhood instance relative to the $i_{th}$ instance and $C(i)=\underset{j}{\sum }w(i,\;j)$. The weight can be computed using Equation 2.

$$\begin{array}{c} w(i,\;j)=\exp\left(-\frac{\sum_{k\in \Delta }{\left(v[i+k]-v[\;j+k]\right)}^{2}}{2L_{\Delta }\mu^{2}}\right)\\ \;=\exp\left(-\frac{d^{2}(v[i],v[j])}{2L_{\Delta }\mu^{2}}\right) \end{array}$$ (2)

Here, μ implies the parameter of the bandwidth, Δ refers to a neighborhood comprising $L_{\Delta }$ instances adjacent to the sample of interest. The term $d^{2}$ denotes the squared Euclidean distance among the set of selected instances, considering the similarity of their neighborhoods. A similar process is repeated for every instance, and we are eventually left with a denoised signal.

In the NLM approach, the instances of clear ECG signals with the same neighborhoods were presumed to be quite close to each other. Consequently, by averaging those instances, the signal is denoised if the noise has a mean value of zero. This presumption is reasonable for digital images, but it cannot apply to the QRS complex in ECG signals. Additionally, the same instances have diverse neighborhoods that frequently specify lower weights. Conversely, the extremely noisy instances have fairly similar neighborhoods and are specified with greater weights. Likewise, in NLM methodology, the calculation of instances does not contribute to the approximation of even adjacent instances.

#### 3.1.2 Detection of R peaks

In QRS regions, the R peaks are positive. They are detected by comparing relative magnitude in every QRS region (25). An exploration for the maximum was completed on relative scales for every window to remove faults owing to standard wander.

1. For every perceived QRS window, the minimum and maximum amplitude values of the ECG data array are computed.
2. From every data point, the mean of maximum and minimum values is deducted to acquire the relative magnitude.
3. The location of maximum relative magnitude is the R-point position of the corresponding QRS window. The complete maximum value of the QRS window is not chosen as the R-point position to remove the likelihood of S recognition.

The mechanism of adaptive thresholding classifies R-peaks as local maxima that are higher than a dynamic threshold as provided in Equation 3:

$$t_{R}=\{t_{i}y(t_{i})>T(t_{i}),\;y(t_{i})\;is\;a\;local\;maximum\}$$ (3)

whereas $\;y(t_{i})$ denotes an improved ECG signal and $T(t_{i})$ means that an adaptive threshold depends upon signal statistics. This ensures strong recognition under fluctuating noise conditions or heart rate variations. The output is a series of accurate R-peak locations employed for analysis of cardiac rhythm.

#### 3.1.3 Segmentation

The final pre-processing phase includes segmentation, which divides the ECG signal into significant intervals for continued classification and feature extraction. Utilizing the detected R-peaks, every ECG cycle (heartbeat) is segmented from a single R-peak to the next, creating RR intervals. These segments can be more aligned and standardized to a fixed length to create a uniform database appropriate for ML or DL systems. Segmentation helps in separating discrete cardiac cycles and also aids in recognizing temporal abnormalities like waveform deformations, missed beats, and arrhythmic patterns.

### 3.2 Two-dimensional spectral representation using STFT

In this step, to utilize the efficacy of the DL algorithms, every pre-processed ECG segment is converted into a two-dimensional representation through STFT to produce spectrograms. In 2D-CNN, the input data are needed for the image type (26). Thus, the ECG signals in the time domain that belong to five kinds of heart disease are primarily transformed into 2D time-frequency spectrograms utilizing STFT. The STFT works to change one-dimensional ECG signals into two-dimensional spectrogram illustrations that at the same time retain temporal and frequency-domain data. Unlike conventional time-domain methods, STFT delivers localized frequency features by applying Fourier analysis to small overlapping signal windows. This demonstration conserves dynamic differences and subtle morphological patterns in ECG signals, allowing DL techniques to efficiently extract discriminative attributes. Additionally, the spectrogram format enables the use of recognized computer vision frameworks, enhancing robustness and reorganization performance under arrhythmic conditions.

STFT is an expansion of the Fourier transform that allows localization of the frequency content of a signal in time. The STFT is evaluated by separating the signal into distinct segments, multiplying every segment by the window function *w* concentrated at time τ and calculating the Fourier transform as provided in Equations 4, 5.

$$\mathrm{STFT}\;(x)(\tau ,\;\omega )=\overset{\infty }{\underset{-\infty }{\int }}x(t)w(t-\tau )e^{-i\omega t}dt$$ (4)

$$\mathrm{STFT}\;(x)(m,\;\omega )=\overset{N}{\underset{n=0}{\sum }}x[n]w[n-m]e^{-i\omega n}$$ (5)

Employing the Hann function for data windowing decreases the likelihood of spectral leakage using Equation 6.

$$w[n]=\frac{1}{2}\left[1-\;\cos\;\left(\frac{2\pi n}{N}\right)\right]$$ (6)

Once the STFT is applied, the spectrogram is obtained to evaluate the ability of spectral elements by the use of Equation 7:

$$\mathrm{Spectrogram}\;(x)\;[m,\;\omega ]=|\overset{N}{\underset{n=0}{\sum }}x[n]w[n-m]e^{-i\omega n}{|}^{2}$$ (7)

Representation of a signal as a spectrogram aids in managing the limitation of the simple Fourier transform, which offers data about the frequency characteristics of the signal without temporal localization. In ECG examination, acquiring changes in frequency through time is significant to recognizing rhythm abnormalities and alterations in frequency elements connected with diverse cardiac events.

### 3.3 Feature extraction via deep learning models

SqueezeNet, NASNet, and EfficientNet are used as feature extractors for the feature extraction process to get diverse and rich representations of the input pest images. Each network learns complementary discriminative characteristics based on its unique framework features. The extracted feature vectors are fused using feature-level concatenation to form a comprehensive and unified representation. This fusion methodology integrates the intensities of all three networks, enhancing feature diversity and discriminative capability and reduces information loss. The feature vector fused is subsequently sent to the classification stage for precise pest identification.

#### 3.3.1 Squeezenet

SqueezeNet decreases the number of parameters by 50 times, or more, reaching an AlexNet-level top-5 error rate for employing FPGA and another resource-scarce model. This model is effective because of its lightweight framework and efficient parameter utilization. It uses Fire components composed of squeeze and expand layers to decrease method complexity while conserving representational ability. This methodology extracts discriminative attributes with significantly fewer parameters and lower computational cost. Furthermore, its compact design reduces overfitting and allows it to learn subtle ECG morphological features. The reduced technique size similarly enables earlier training and inference without compromising feature extraction excellence. To achieve this, three key approaches are utilized: transforming 3×3 convolutional filters into 1×1 filters that require nine times fewer parameters; diminishing the count of input to 3×3 filters by the layer of squeeze; and achieving down-sampling at subsequent phases of the network by upholding the higher spatial resolution of attributes in the initial layer, thus improving the performance of the model. SqueezeNet has developed a module called the fire module (27). The objective of the fire module is to create more effective parameters than in other techniques. It comprises two major elements: a layer of squeeze with 1×1 filters lessening the number of input channels and an expanding layer integrating 1×1 and 3×3 filters by regaining the intricacy for removal of features. This framework enables the fire module by striking the right balance between decreasing parameters and maintaining computational efficiency, as the boundaries of the squeeze layer and the dimension of the input to expand the layer, thus reducing the overall parameter counts. Consequently, the fire module allows for a more adaptable but slim model for an effective and dependable CNN, featuring selectively tuned squeeze and expanding layer structures.

**Figure 2:**
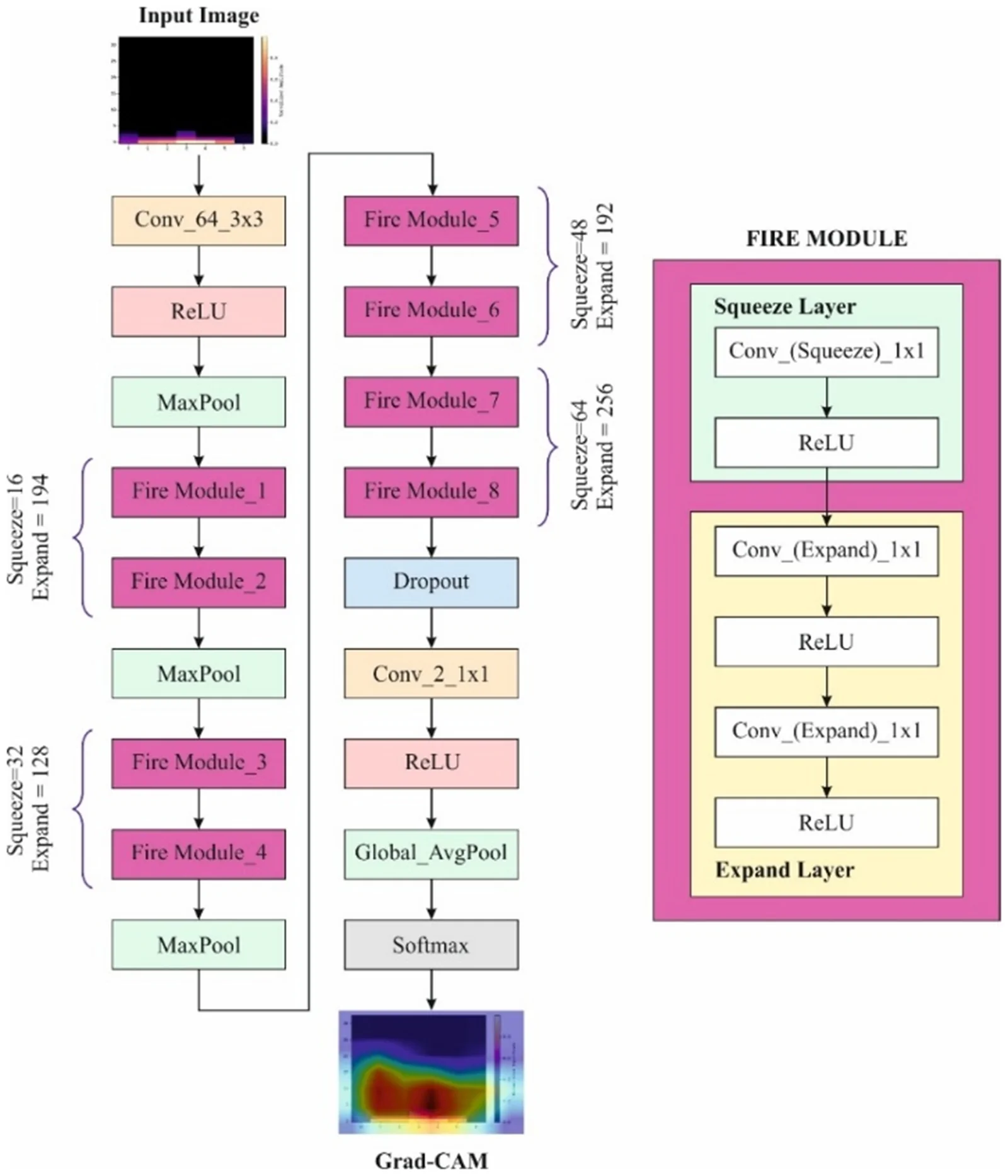
General process of SqueezeNet architecture.

SqueezeNet starts with a primary convolution layer (conv1), followed by eight fire layers (fire2 to fire9), and subsequently an ending convolution layer (conv10). The no. of filters in every fire module progressively increases from the initial to the final phase. Max-pooling has been accomplished in each of the dual layers after conv1, fire4, fire8, and conv10. Figure 2 depicts the general process of the SqueezeNet architecture.

The SqueezeNet framework has been applied with various model selections and optimizers to improve its outcomes.

- In expanding layers, a single-pixel zero-padding border has been added to the input of the3×3 filter by ensuring that the outcome activation from 1×1 and 3×3 filters holds similar dimensions.
- The activation of ReLU is applied to the outcomes from both the squeeze and expanding layers, which gives non-linearity.
- A dropout layer with a rate of 50% is implemented after the final fire module (fire9) to decrease overfitting and improve the generality of the model.
- The frameworks avoid fully connected layers, derived from the NiN model by diminishing the number of parameters and improving efficacy.
- Training begins with a learning rate that decreases linearly over time.
- Owing to the limitations of the model, the expanding layer employs two dissimilar convolution layers, like 1×1 filters and 3×3 filters, and their outcomes are concatenated along the channel.
- Initially developed with the Caffe framework, SqueezeNet is suited for platforms, namely Chainer, MXNet, Torch, and Keras, as it ensures extensive compatibility.

#### 3.3.2 NASNet

The NASNet automates the DL approach for classification. NASNet leverages a two-stage search: a controller that creates a framework and a searching area describing probable parameters and structures (28). NASNet is combined because it uses Neural Architecture Search (NAS) to instinctively realize optimal network frameworks. The algorithm proficiently learns hierarchical and compound attribute illustrations that could not be processed by manually considered systems. Its cell-based method increases flexibility and allows extraction of varied ECG patterns under changing signal conditions. Besides, NASNet improves feature learning ability by recognizing methodology configurations enhanced for classification outcome and generalization. In the primary stage, reinforcement learning discovers alternative frameworks, and in another stage, these frameworks are used to calculate the outcome of validation data. The best-performing framework is then trained for classification. The structure of NASNet comprises cells classified as reduction and normal cells. The output of every cell is processed by pooling, convolution, and activation functions. The output *y* of the NASNet cell is specified using Equation 8:

$$y=\overset{n}{\underset{i=1}{\sum }}w_{i}.\sigma \left(\overset{m}{\underset{\;j=1}{\sum }}(g_{j}\circ f_{j}(x)\right).W_{i,j})+b$$ (8)

Where σ signifies a non-linear activation function, *b* refers to a biased term, $g_{j}$ denotes various operations employed to input x, and $W_{i,j}$ are learnable weights connecting inputs to outputs, which can be represented using Equation 9.

$$L(\theta )=\frac{1}{N}\overset{N}{\underset{i=1}{\sum }}(y_{i}-\hat{y_{l}}{)}^{2}+\lambda \overset{M}{\underset{\;j=1}{\sum }}\|\theta_{j}{\|}^{2}$$ (9)

Now $\hat{y_{1}}$ signifies the predicted outcome, $y_{i}$ denotes the true label, λ implies the regularization parameter, *N* represents the counts of samples, $\|\theta_{j}{\|}^{2}$ refers to the L2 norm of parameters to uphold overfitting, and *M* depicts the overall parameter counts.

#### 3.3.3 Efficientnet

The EfficientNet network is a lightweight DL model. Unlike classical neural network frameworks that improve performance by independently modifying channel width, network depth, and resolution of the input image, this method presents a composite scaling approach (29). The EfficientNet is used due to its complex scaling algorithm that analytically balances network width, depth, and input resolution. This approach allows well-organized extraction of rich and fine-grained attributes while maintaining inferior computational requirements. EfficientNet increases feature illustration over balanced scaling instead of random network expansion. Furthermore, its optimized technique gives maximum accuracy–efficacy trade-offs and improves robustness under signal variability and arrhythmic conditions. A mixing factor, signified as φ, uniformly scales the depth, width, and resolution parameters. The computation for ϕ is as follows:

$$\begin{array}{c} depth:d=\alpha^{\varphi }\\ width:\;w=\beta^{\varphi }\\ resolution:\;r=\gamma^{\varphi }\\ s.t.\;\;\;\;\;\alpha \cdot \beta^{2}\cdot \gamma^{2}\approx 2\\ \alpha \geq 1,\;\beta \geq 1,\;\gamma \geq 1 \end{array}$$ (10)

**Figure 3:**
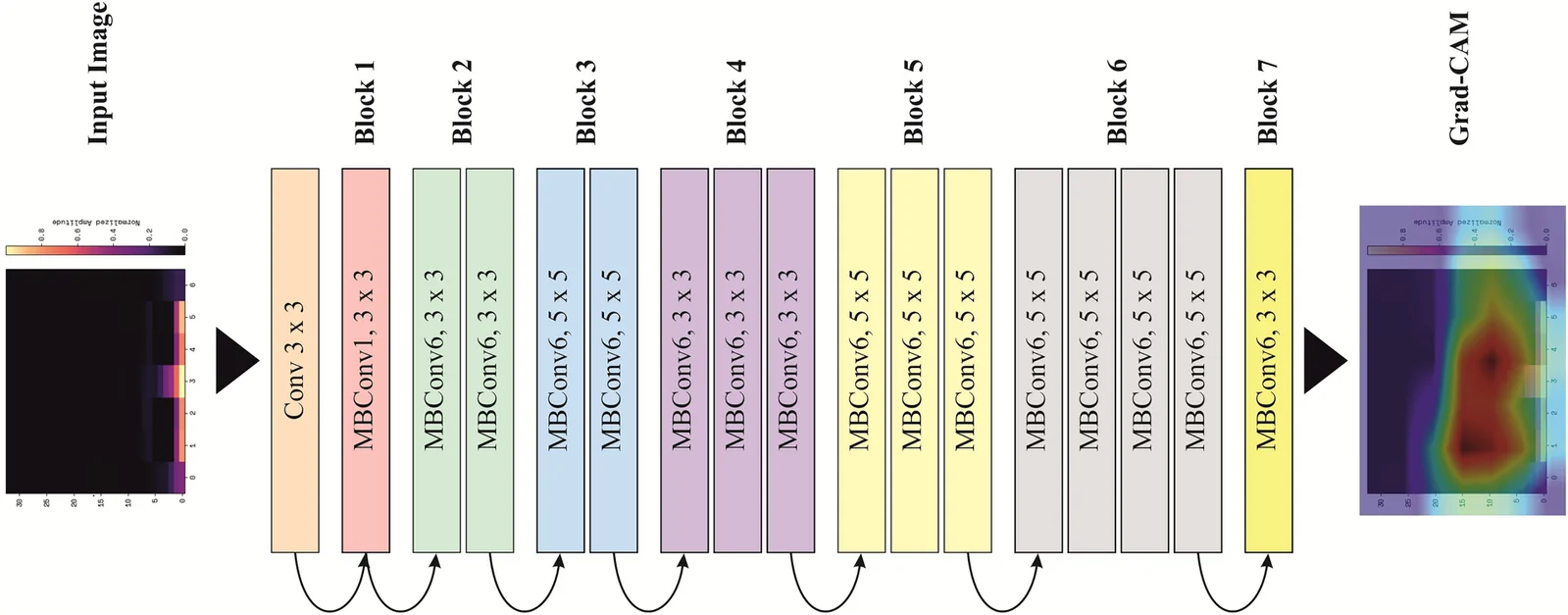
Architecture of EfficientNet.

Now φ refers to scale mixing factor; β, and γ are the equivalent scaling parameters; *d*, *w*, and *r* refer to width, depth, and resolution. While ϕ=1, the most lightweight approach, EfficientNet-B0, begins by integrating Equation 10 with the MNAS (Automated Mobile Neural Architecture Search) model, employing scaling factors of α=1.2, β=1.1, and γ=1.15. According to these three proportional coefficients, the Bl to B7 network is attained by incrementally varying the mixture coefficient ϕ. EfficientNet-B0 was used as the benchmark model owing to its relatively smaller number of parameters. Figure 3 illustrates the architecture of EfficientNet.

### 3.4 Proposed Siamese-LSTM autoencoder architecture

This section introduces the proposed Siamese LSTM-based autoencoder, termed the Siamese Recurrent Autoencoder (SRAE), which serves as the core architecture for the classification framework.

#### 3.4.1 Auto encoders (AEs)

AE extensively utilized the DL approach. As a specific form of NN, AEs utilize an encoder to attain a representation by converting original data into a latent space and subsequently leveragomg a decoder to reconstruct data with latent space (30).

z=f(wx+b) (11)

$$\tilde{x}=f(w^{\prime }z+b^{\prime })$$ (12)

In Equations 11 and 12, $x,z,\tilde{x}$ signify input data, latent attributes eliminated by the encoder, and the reconstruction outcomes attained by decoder, respectively. *f* denotes non-linear representation of the decoder and encoder. *w* and $w^{\prime }$ are the weight and *b* and $b^{\prime }$ denote bias. The training targets of AE are optimized to lessen the reconstruction error between *x* and $\tilde{x}$. The reconstruction error has been typically utilized as an indicator in the AE-based approach. To enhance the outcome, the framework of NN in AE must be altered based on the data features of diverse processes.

#### 3.4.2 LSTM neural networks

LSTM is a renowned type of RNNs, eliminating long-term time dependency. RNNs are usually employed to acquire the process by modifying hidden states. Nevertheless, as the number of timesteps rises, traditional RNNs struggle with long-term dependencies. It strengthens memory transitions regulated by gated architecture.

$$i_{t}=\sigma (W_{i}\cdot [h_{t-1},x_{t}]+b_{i})$$ (13)

$$f_{c}=\sigma (W_{f}\cdot [h_{t-1},x_{t}]+b_{f})$$ (14)

$$g_{t}=ranh(W_{g}\cdot [h_{t-1},x_{\tau }]+b_{g})$$ (15)

$$o_{t}=\sigma (W_{o}\cdot [h_{t-1},x_{t}]+b_{o})$$ (16)

$$C_{t}=f_{t}⊙C_{t-1}+i_{\tau }⊙g_{t}$$ (17)

$$h_{t}=o_{t}⊙\;\tanh\;(C_{t})$$ (18)

In Equations 13–18, gt denotes the existing state of the candidate, xt represents the input at the existing time step, $i_{t}$, $f_{t}$, $o_{t}$ are the input, forget, and output gates, respectively, $h_{t}$, $h_{t-\;1}$ refer to concealed states of the existing and final time-step, $C_{t}$, $C_{t-1}$ depict the memory cell of the present and final time-step, σ, tanh are the sigmoid and tanh activation functions, and *W*, *b* denote the weight and bias. In the gating framework, irrelevant data of the hidden state from the preceding time-step are forgotten, and beneficial data from the existing time step are retained in the memory cell, meaning the LSTM component is efficient in eliminating long-term time dependency.

#### 3.4.3 Siamese neural network (SNN)

The SNN is a kind of NN framework that comprises two parallel NNs that share a similar framework and weight. SNN is trained together on a pair of input data by comparing their similarity or learning the relationship between them. It exhibits substantial capability for few-shot learning or even one-shot learning to resolve the confirmation of an image.

SNN comprises dual parallel networks. All colored bars are considered latent representations attained from distinct but similar NNs in the structure of a Siamese network. They are parallel and process data individually, but they share a similar fundamental parameter and structure. A special feature in every Siamese network can receive a diverse portion of data as input. The similarity between dual inputs is determined by the target function, typically the Euclidean distance measure of similarity or Kullback-Leibler divergence, by calculating the similarity of their distribution.

If the diverse portions of input data originate from numerous resources and show various methods but reveal certain connections, the weights and structures of the Siamese networks are dissimilar when acquiring latent attributes from diverse portions of input data. The methodology is called a pseudo-Siamese network. The SNN method shares a similar structure and weight, as both inputs are made up of similar kinds of data.

#### 3.4.4 Design of the SRAE

To enhance the generality of DL-based approaches, an innovative SRAE method is presented using Equations 19 and 20:

$$z_{1}=LSTM(x_{1})$$ (19)

$$z_{2}=LSTM(x_{2})$$ (20)

Here, $z_{1},z_{2}$ implies equivalent latent features eliminated by the encoder of LSTM and $\chi_{1},\chi_{2}$ refers to dual inputs of SNN. Consequently, the dual inputs have similar resources. Thus, the SNN method in the presented SRAE shares a similar structure and weight. Conversely, the generalizability of the method is trained with a similar sample size. To specify *n* training instances, the sample size is extended to n(n−1)/2 by SNN with binary inputs.

The process of training data for NN input substantially impacts the performance. Data shuffling is an essential feature of NN training in numerous contexts, and it improves data independence and ability to generalize and possibly avoids local minima. In such cases, the upcoming values frequently rely on past values. Shuffling disrupts this dependency; it makes the method complex by acquiring the temporal patterns and dynamic processes. Thus, it maintains the original order of data gathered under normal conditions, assuring that the method can effectively learn and utilize this temporal dependency. To specify normal conditions, the separation of $x_{1}$ and $x_{2}$ are inputs.

The SNN necessitates dual inputs, whereas the essential feature of data exists in temporal sequence relations among its data points. It is vital to ensure the sequential relation remains as complete as possible. The dual input of SNN uses diverse segments from similar data. It adopts the first half of the data function as primary input $\chi_{1}$ and the other half as secondary input $x_{2}$. While there is a temporal variance among dual inputs, they both belong to the normal condition. To employ the SNN and contrastive loss, their depictions in feature space are similar or nearly aligned, thereby ensuring efficacy.

Furthermore, a contrastive loss function is used to regularize the distribution of hidden attributes.

$$loss=w_{1}\frac{\left\|\right\|x_{1}-\tilde{x_{1}}{\|}_{2}^{2}}{n}+w_{2}\frac{\left\|\right\|x_{2}-\tilde{x_{2}}{\|}_{2}^{2}}{n}+w_{3}z_{1}-z_{2},\chi_{1},\chi_{2}\in P$$ (21)

$$loss=w_{1}\frac{\left\|\right\|x_{1}-\tilde{x_{1}}{\|}_{2}^{2}}{n}+w_{2}(\max(margin-z_{1}-z_{2},\;0)),\chi_{1}\in P,x_{2}\in N$$ (22)

Here, $w_{1,}w_{2,}w_{3}$ represents the weight of every term of loss function, $\tilde{x_{1}},\tilde{x_{2}}$ refers to the reconstruction of the decoder, *P* indicates input belonging to normal conditions, margin denotes a user-defined parameter, and *N* depicts input belonging to abnormal conditions.

The present AE-based models only reconstruct the reconstruction error by adopting training targets and indicators. The eliminated latent attributes are easily accessible for attaining enhanced reconstruction of original data by the model. In this instance, it is an efficient and reasonable choice for realizing the process instantly. Thus, the loss function in Equations 21, 22 is defined by the latent attributes of dual inputs of similar classes to one another, whereas the latent attributes of dual inputs of diverse classes are less similar. The Euclidean distance has been leveraged to calculate the similarity among latent attributes. The MSE between the input and outcome of the AE is retained to eliminate more descriptive attributes. The prevalence of every item is modified by weights. The latent attributes are eliminated; normal training data can be normalized into a smaller region, and $T^{2}$ statistics were determined by Equation 23.

$$T^{2}=z_{c}\Lambda_{z}^{-1}z_{c}^{T}$$ (23)

Here, $\Lambda_{z}$ represents the covariance matrix of eigenvectors and $z_{f}$ denotes that the latent attributes are eliminated at step *t*. A control limit is set using the Kernel Density Estimation (KDE) approach. As a non-parametric estimation model, KDE offers a trustworthy threshold estimation without necessitating any presumptions about the fundamental distribution of data. The statistics $T^{2}(x)$ are evaluated for normal conditions using Equations 24 and 25:

$$\rho (T^{2}(x))=\frac{1}{\sqrt{2\pi }dr}\overset{t}{\underset{t-1}{\sum }}e^{-\frac{{\left(\tau^{2}(x)-T^{2}(x_{i})\right)}^{2}}{2d^{2}}}$$ (24)

$$\overset{threshold}{\underset{-\infty }{\int }}\rho (T^{2}(x))dT^{2}(x)=0.99$$ (25)

Here, *d* signifies the window width of the Gaussian kernel function utilized. Finally, the process includes three output branches: one for person identification, one for age prediction, and one for gender classification.

The model is trained with adaptive learning using the Nadam optimizer, guaranteeing faster convergence and better generalization. Adaptive learning is a main concept in DL that allows a system to adaptively alter its learning rate at the time of training depending on gradient information (31). Nadam Optimizer is one of the most effective adaptive learning algorithms; it integrates the power of Adam (Adaptive Moment Estimation) and Nesterov Accelerated Gradient (NAG) approaches. The fundamental principle of this algorithm involves the dynamic adjustment of the decay factor β. To enhance accuracy, a series of individual parameters, $\beta_{1},\beta_{2},\ldots ,\beta_{t}$, are evaluated at each step, 1,2⋯,t, as the algorithm alternates between maximizing and minimizing the factor. The application of the momentum in step t+l is used when updating the step t rather than t+l, as represented in Equations 26–30:

$$g_{t}\leftarrow \nabla_{\theta_{t-1}}J_{t}(\theta_{t-1})$$ (26)

$$m_{t}\leftarrow \beta_{t}m_{t-1}+\eta g_{t}$$ (27)

$$\theta_{t}\leftarrow \theta_{t-1}-(\beta_{t+1}m_{t}+\eta g_{t})$$ (28)

$$\theta_{t}\leftarrow \theta_{t-1}-\eta \left[\frac{\beta_{t}m_{t-1}}{1-\prod_{i=1}^{t}\beta_{i}}+\frac{(1-\beta_{t})g_{t}}{1-\prod_{i=1}^{t}\beta_{i}}\right]$$ (29)

$$\theta_{t}\leftarrow \theta_{t-1}-\eta \left[\frac{\beta_{t+1}m_{t}}{1-\prod_{i=1}^{t+1}\beta_{i}}+\frac{(1-\beta_{t})g_{t}}{1-\prod_{i=1}^{t}\beta_{i}}\right]$$ (30)

The proposed architecture employs a hybrid loss function that combines contrastive loss and reconstruction loss for improving the quality of latent feature learning. The reconstruction component reduces the difference between the input ECG feature representation and its output from the decoder, which ensures effective encoding of ECG traits. The contrastive element enforces discriminative learning through minimization of the distance between same ECG pairs and maximization of the distance between diverse pairs.

## 4 Results and discussions

**Figure 4:**
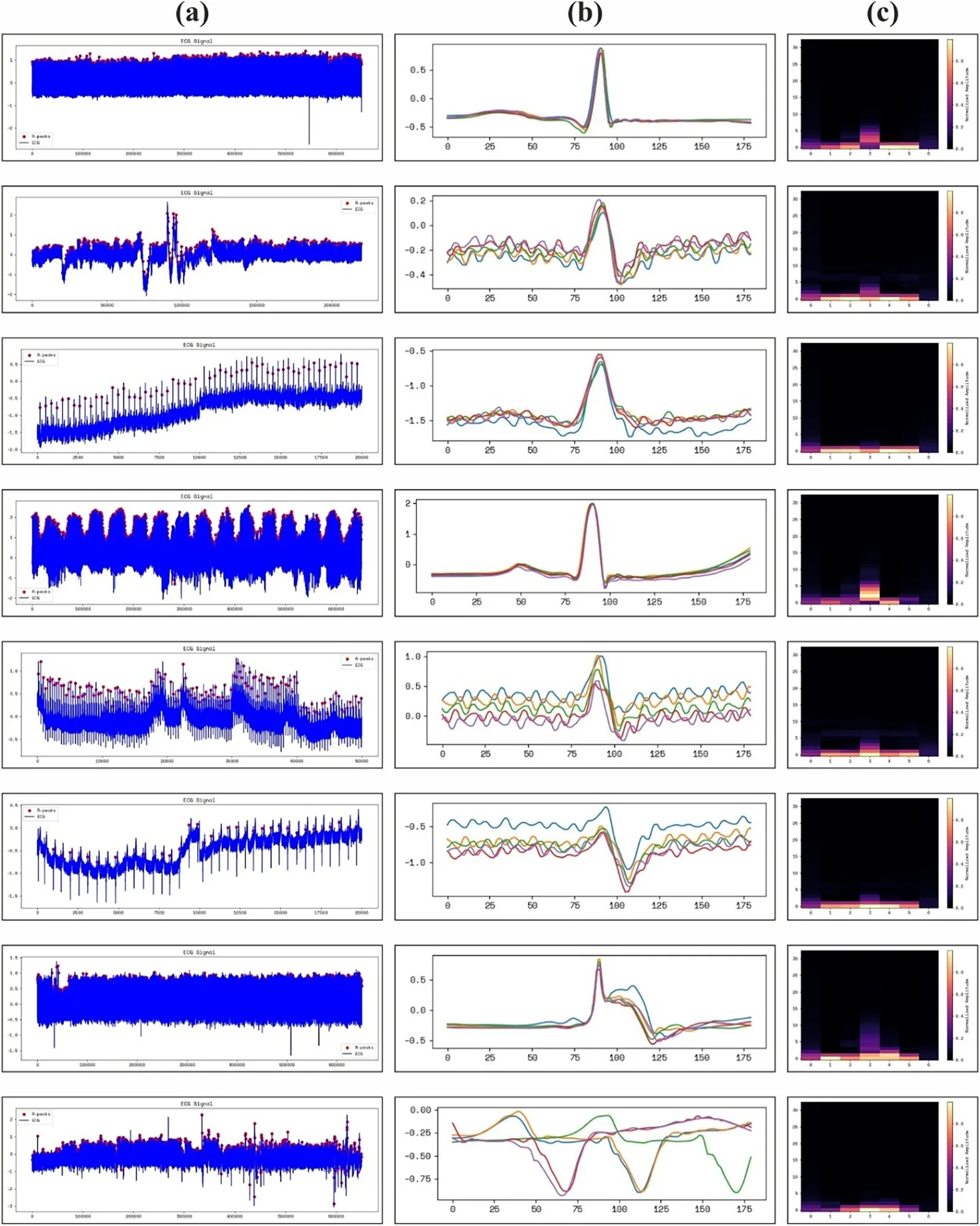
Sample images **(a)** ECG, **(b)** R-peak segmented, and **(c)** spectrograms.

The proposed model is simulated using the Python 3.11.9 tool on a PC i5-8600k, GeForce 1050Ti 4GB, 16GB RAM, 250GB SSD, and 1TB HDD. In this section, the experimental analysis of the FDDR-ID system is verified under the MIT-BIH (32) and ECG-ID (33) datasets. For experimental validation, 80% of the training and 20% of the testing dataset are used. Figure 4 represents the sample images of the ECG signal, R-peak segmented, and spectrogram. Table 2 represents the Parameter settings of the proposed methodology. In this setting, the model is trained for 128 epochs with a batch size of 32, meaning weights are updated after every 32 samples, and the training runs over 128 complete passes through the dataset. The learning rate of 0.0001 guarantees precise and gradual updates to the model weights, assisting in preventing overshooting and enhancing accuracy.

**Table 2:** Parameter setting.

Method	Parameter	Value
ECG Denoising	Filter	Non-Local Means (NLM)
R-Peak Detection	Threshold	Adaptive
STFT	Window Length	256
STFT	Overlap	0.5
Feature Extraction	SqueezeNet	Fire Modules (8)
Feature Extraction	NASNet	NASNet-Mobile
Feature Extraction	EfficientNet	EfficientNet-B0
Feature Fusion	Embedding Size	512
Siamese LSTM Autoencoder	Hidden Units	256
Activation	Function	ReLU
Optimizer	Algorithm	Nadam
Learning Rate	Initial LR	0.01
Dropout	Rate	0.5
Batch Size	Batch Size	5
Training	Epochs	50

### 4.1 Result analysis of MIT-BIH dataset

The experimental analysis was conducted utilizing the MIT-BIH Arrhythmia Database, which is publicly accessible on the PhysioNet repository, and the ECG-ID Database for experimental assessments. Initially, the ECG recordings are preprocessed using NLM filtering for noise removal, followed by heartbeat localization by R-peak detection. The individual heartbeat sections were extracted over each detected R-peak and converted into spectrogram images by STFT. The samples segmented were then allocated labels based on the corresponding subjects' age, gender, and identity information, which is available in the corresponding datasets. To ensure balanced training, an equal number of samples were used from the classes after preprocessing and segmentation. This balanced dataset was consequently used for training and testing the given multitask architecture. The dataset contains 48 ECG recordings, each signifying various cardiac states and patient variations. All classes consist of 60 ECG signal examples, leading to a total of 2,880 examples employed for training and validation. The recordings were originally acquired at a sampling frequency of 360 Hz from 48 subjects, with expert annotations for every heartbeat type. This dataset offers higher-quality, clinically verified ECG signals that are broadly utilized for R-peak identification, cardiac rhythm classification, and arrhythmia detection. Its balanced composition and comprehensive labelling make it an ideal benchmark to evaluate DL models and ECG signal processing.

**Figure 5:**
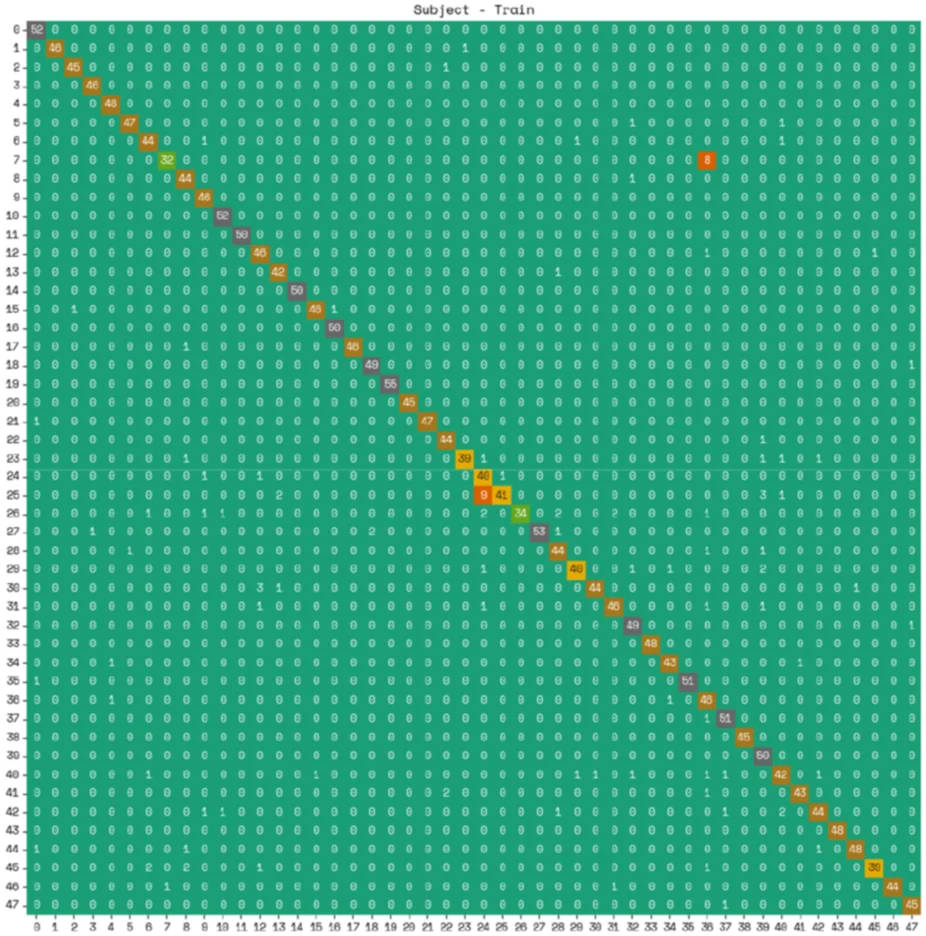
Confusion matrix for person identification under the MIT-BIH dataset.

Figure 5 shows the confusion matrix for person identification under the MIT-BIH dataset. The result indicates that the FDDR-ID approach shows effective identification and detection of each class accurately.

Table 3 shows the training and testing set outcomes of the FDDR-ID model for person identification using the MIT-BIH dataset. On the training set, the proposed FDDR-ID model has achieved average $accur_{y}$, $preci_{n}$, $recal_{l},\;F1_{score}$, and MCC values of of 95.01%, 95.33%, 94.97%, 94.95%, and 94.91%, respectively. Also, on the testing set, the proposed FDDR-ID technique has attained average $accur_{y}$, $preci_{n}$, $recal_{l},\;F1_{score}$, and MCC values of 78.13%, 79.41%, 79.75%, 78.04%, and 77.70%, respectively.

**Table 3:** Training and testing sets outcome for person identification under the MIT-BIH dataset.

Person Identification		
Metrics	Training Set	Testing Set
Accuracy	95.01	78.13
Precision	95.33	79.41
Recall	94.97	79.75
F1-Score	94.95	78.04
MCC	94.91	77.70

**Figure 6:**
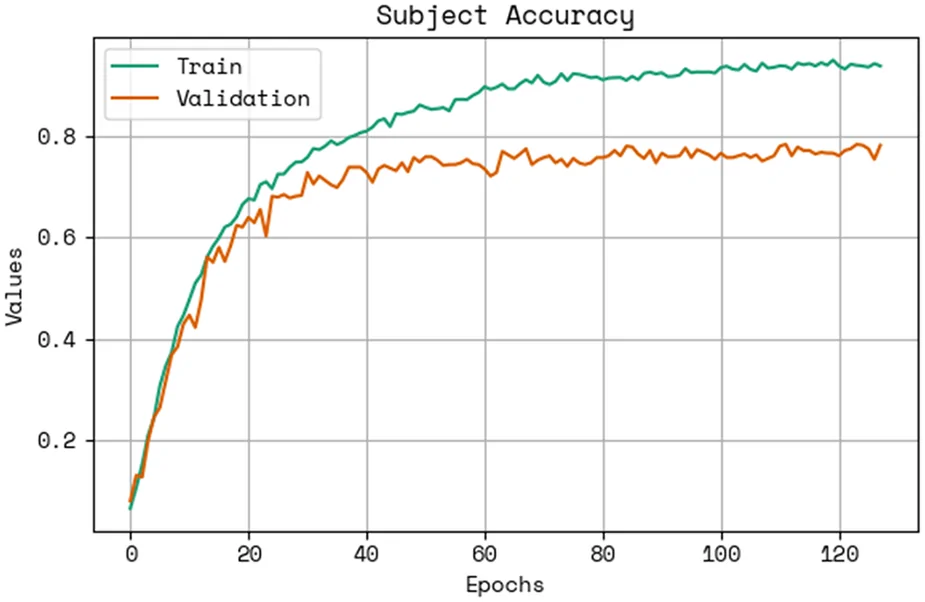
Accuracy curve for person identification under the MIT-BIH dataset.

Figure 6 shows the training (TRAN) and validation (VALD) accuracy of the FDDR-ID method for person identification under the MIT-BIH dataset over 120 epochs. Both curves progressively rise and slowly converge, which indicates that the model is learning efficiently. The VALD accuracy constantly remains slightly superior to the TRAN accuracy, implying that the model is not overfitting and generalizes well to unseen data. The fluctuations in accuracy are expected because of the difficulty of the task, but the overall upward tendency indicates stronger performance and steadiness of the model.

**Figure 7:**
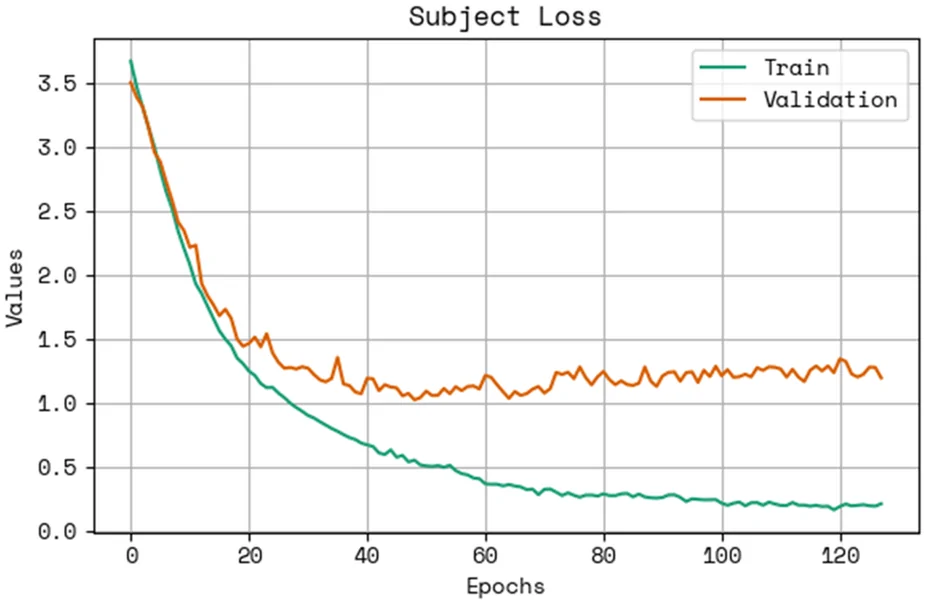
Loss curve for person identification under the MIT-BIH dataset.

Figure 7 shows the TRAN and VALD loss of the FDDR-ID model for person identification under the MIT-BIH dataset over 120 epochs. Both curves display a constant downward trend, signifying that the model effectively minimizes error in learning. The VALD loss remains slightly inferior to the training loss through most epochs, implying good generalization and no signs of overfitting. Though some fluctuations are observed, they become steadier and more consistent.

Table 4 shows the training and testing set outcomes of the FDDR-ID technique for age prediction under the MIT-BIH dataset. Under the training set, the proposed FDDR-ID model has obtained the following average values: MSE of 5.791034, RMSE of 2.406457, MAE of 0.449954, and R2-Score of 0.984813. Also, under the testing set, the proposed FDDR-ID model has obtained the following average values: MSE of 38.50868, RMSE of 6.205536, MAE of 1.206913, and R2-Score of 0.90658.

**Table 4:** Training and testing sets outcome for age prediction under the MIT-BIH dataset.

Age Prediction		
Metrics	Training Set	Testing Set
MSE	5.791034	38.50868
RMSE	2.406457	6.205536
MAE	0.449954	1.206913
R2-Score	0.984813	0.90658

**Figure 8:**
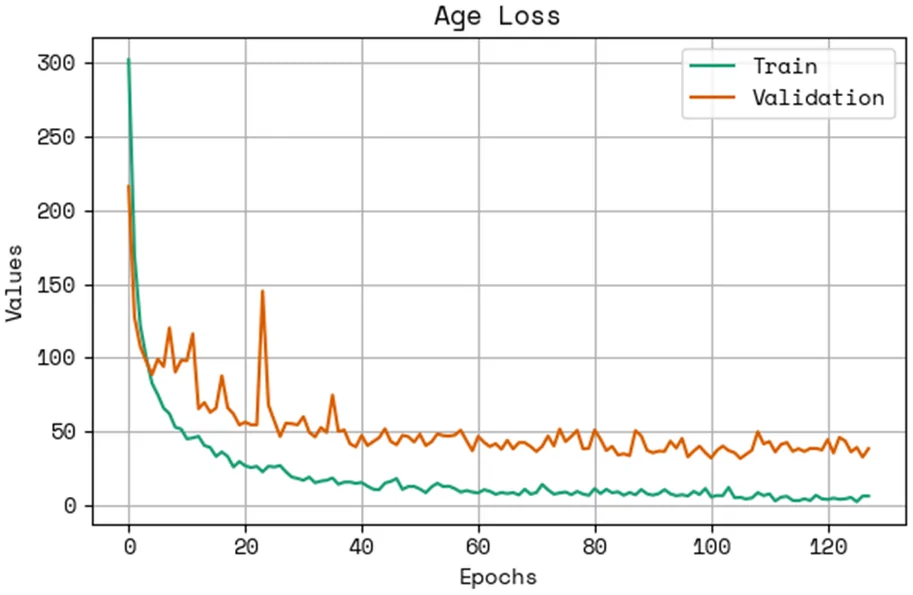
Loss curve for age prediction under the MIT-BIH dataset.

Figure 8 shows the TRAN and VALD loss of the FDDR-ID method for age prediction under the MIT-BIH dataset over 120 epochs. Both curves display a constant downward tendency, signifying that the model effectively minimizes error throughout learning. The VALD loss stays slightly lower than the training loss over most epochs, implying good generalization and no signs of overfitting. Though some fluctuations are observed, it is becoming progressively more consistent and steady.

Table 5 shows the training and testing set outcomes of the FDDR-ID model for gender identification under the MIT-BIH dataset. In terms of the training set, the proposed FDDR-ID model has reached average $accur_{y}$, $preci_{n}$, $recal_{l},\;F1_{score}$, and MCC values of 99.91%, 99.47%, 99.47%, 99.47%, and 98.93%, respectively. Also, for the testing set, the proposed FDDR-ID model has attained average $accur_{y}$, $preci_{n}$, $recal_{l},\;F1_{score}$, and MCC values of 99.83%, 99.91%, 97.73%, 98.79%, and 97.61%, respectively.

**Table 5:** Training and testing sets outcome for gender identification under MIT-BIH dataset.

Gender Identification		
Metrics	Training Set	Testing Set
Accuracy	99.91	99.83
Precision	99.47	99.91
Recall	99.47	97.73
F1-Score	99.47	98.79
MCC	98.93	97.61

**Figure 9:**
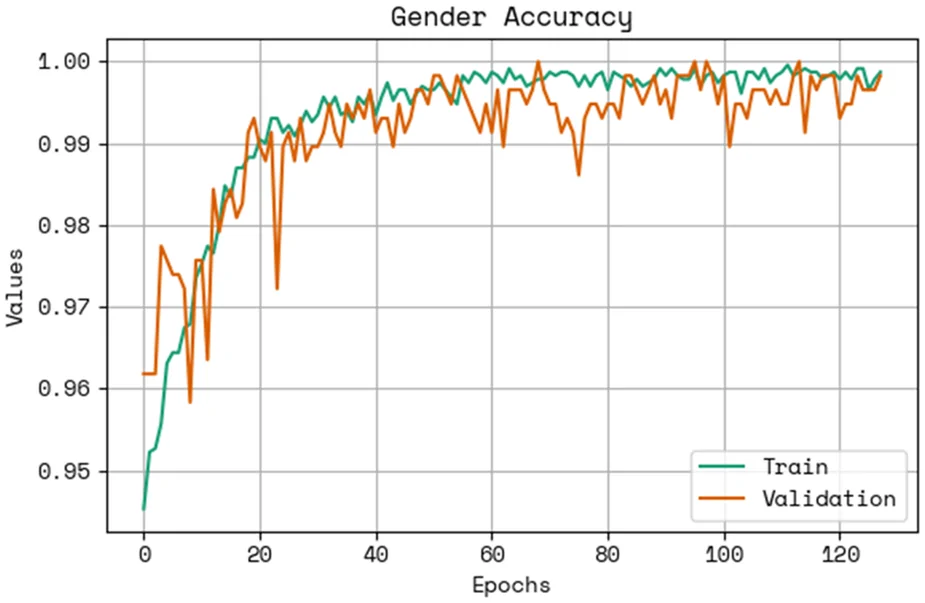
Accuracy curve for gender identification under the MIT-BIH dataset.

Figure 9 shows the TRAN and VALD accuracy of the FDDR-ID model for gender identification under the MIT-BIH dataset over 120 epochs. Both curves gradually rise and slowly converge, which shows that the model is learning efficiently. The VALD accuracy constantly remains slightly greater than the TRAN accuracy, implying that the model is not overfitting and generalizes well to unseen data. The fluctuations in accuracy are expected due to the intricacy of the task, but the overall upward tendency indicates stronger performance and highlihgts the steadiness of the model.

**Figure 10:**
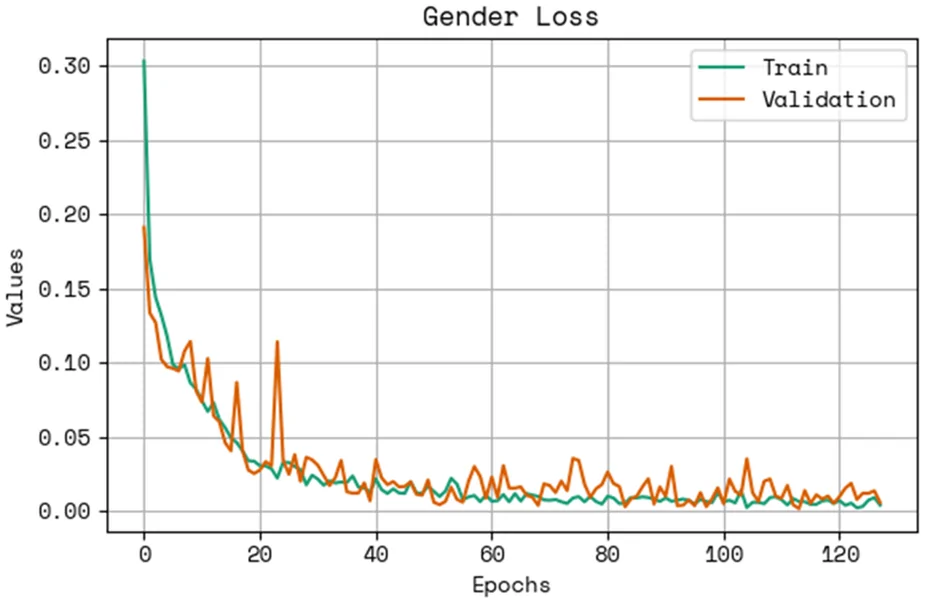
Loss curve for gender identification under the MIT-BIH dataset.

Figure 10 shows the TRAN and VALD loss of the FDDR-ID model for gender identification under the MIT-BIH dataset over 120 epochs. Both curves display a constant downward tendency, representing that the model efficiently minimizes error in learning. The VALD loss remains slightly lower than the training loss through most epochs, implying good generalization and no signs of overfitting. Even though some fluctuations are observed, it is becoming gradually more reliable and stable.

**Figure 11:**
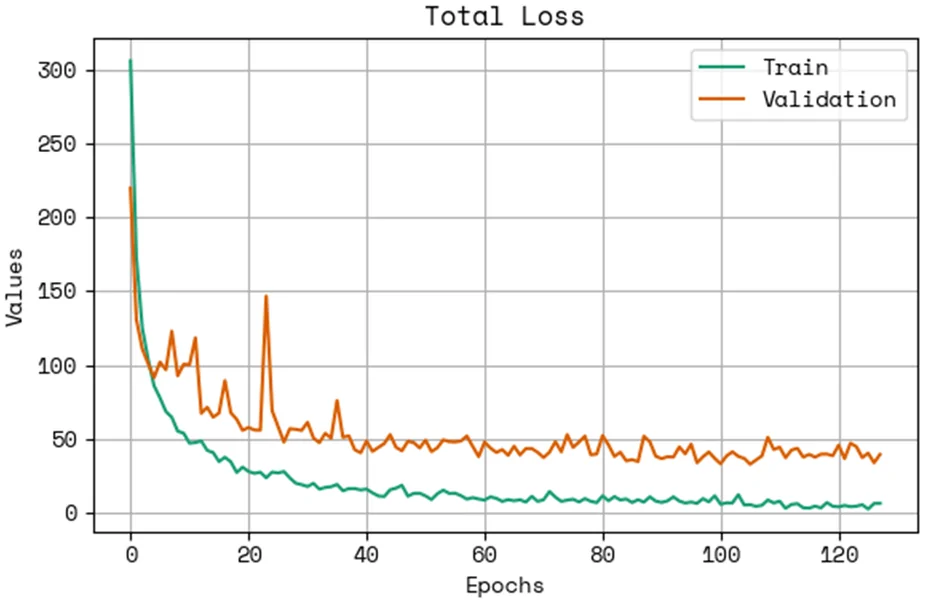
Total loss curve for person identification, age prediction, and gender identification under the MIT-BIH dataset.

Figure 11 shows the TRAN and VALD loss for person identification, age prediction, and gender identification under the MIT-BIH dataset over a sequence of epochs throughout model training. The green line signifies the training loss, which reduces gradually and stabilizes at a lower value, signifying that the model is learning effectively using the training data. The orange line displays the validation loss, which primarily reduces but then fluctuates and remains higher than the training loss. This gap between the two losses indicates overfitting, meaning the model is relatively successful when used on the training data but struggles to generalize to unseen validation data. Overall, while training convergence is obtained, additional models like regularization, early stopping, or dropout can be required to enhance generalization performance.

### 4.2 Result analysis of ECG-ID dataset

The ECG-ID Database contains 310 ECG recordings, obtained from 90 persons. So, we get 90 classes; each class has 60 samples. The total number of samples is 5,400, but our total is 4,972, because the ECG signal has many more samples, so we get our convenient samples. ECG lead I, recorded for 20 s and digitized at 500 Hz with 12-bit resolution over a nominal ±10 mV range. There are a total of 10 annotated beats (unaudited R- and T-wave peak annotations from an automated detector). The ECG-ID database has ECG recordings from various subjects under resting conditions. First, the ECG signals were pre-processed using Non-Local Means (NLM) filtering to reduce noise while preserving the important signal features. Consequently, R-peaks were detected, and separate heartbeat segments were extracted by selecting a fixed-length window that is centered on each detected R-peak. These heartbeat signals were then sent to spectrogram images by the STFT. Poor signal quality segments and missing R-peaks were excluded from further analysis. After labeling, quality screening, segmentation, and preprocessing, a total of 4,972 valid heartbeat specimens were obtained and utilized for training and evaluation.

**Figure 12:**
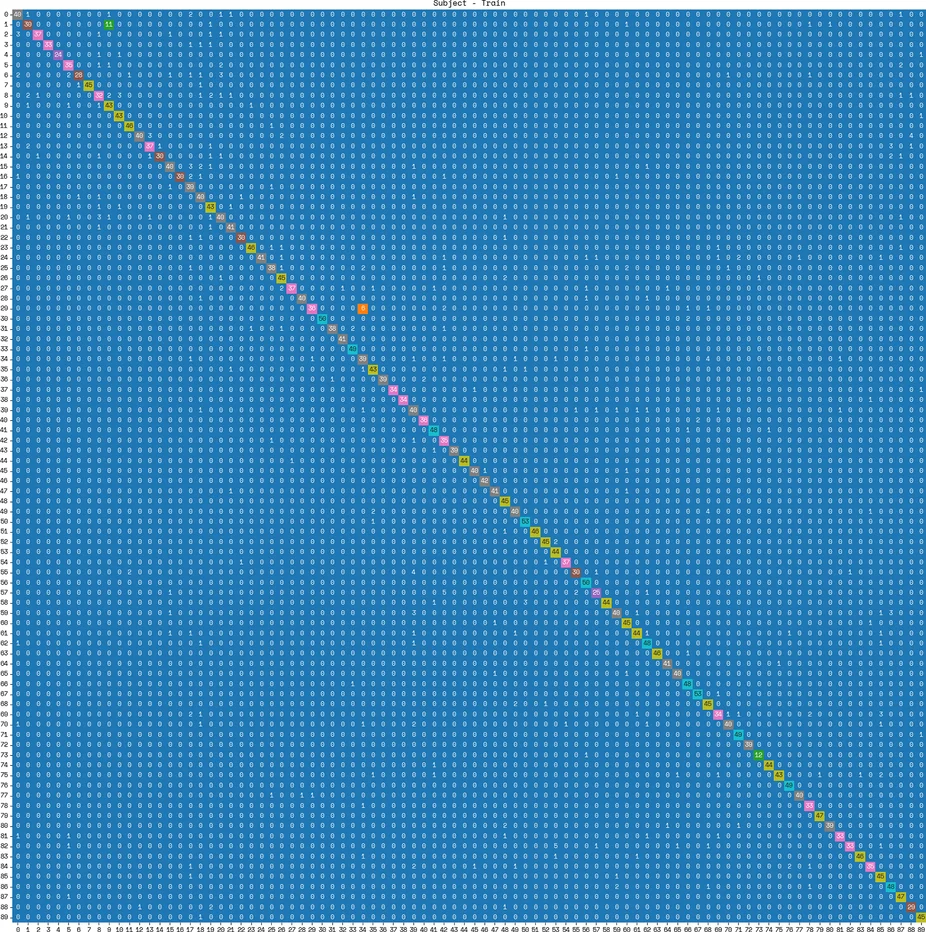
Confusion matrix for person identification under the ECG-ID dataset.

Figure 12 shows the confusion matrix for person identification under the ECG-ID dataset. The results imply that the FDDR-ID approach is effective for accurate identification and detection of every class.

Table 6 shows the training and testing set outcomes of the FDDR-ID technique for person identification under the ECG-ID dataset. On the training set, the proposed FDDR-ID approach has accomplished average $accur_{acy}$, $preci_{sion}$, $recal_{l},\;F1_{score}$, and MCC values of 90.52%, 91.22%, 90.47%, 90.57%, and 90.42%, respectively. In addition, on the testing set, the proposed FDDR-ID method gained average $accur_{y}$, $preci_{n}$, $recal_{l},\;F1_{score}$, and MCC values of 56.28%, 57.99%, 56.62%, 55.50%, and 55.81%, respectively.

**Table 6:** Training and testing sets outcome for person identification under ECG-ID dataset.

Person Identification		
Metrics	Training Set	Testing Set
Accuracy	90.52	56.28
Precision	91.22	57.99
Recall	90.47	56.62
F1-Score	90.57	55.50
MCC	90.42	55.81

**Figure 13:**
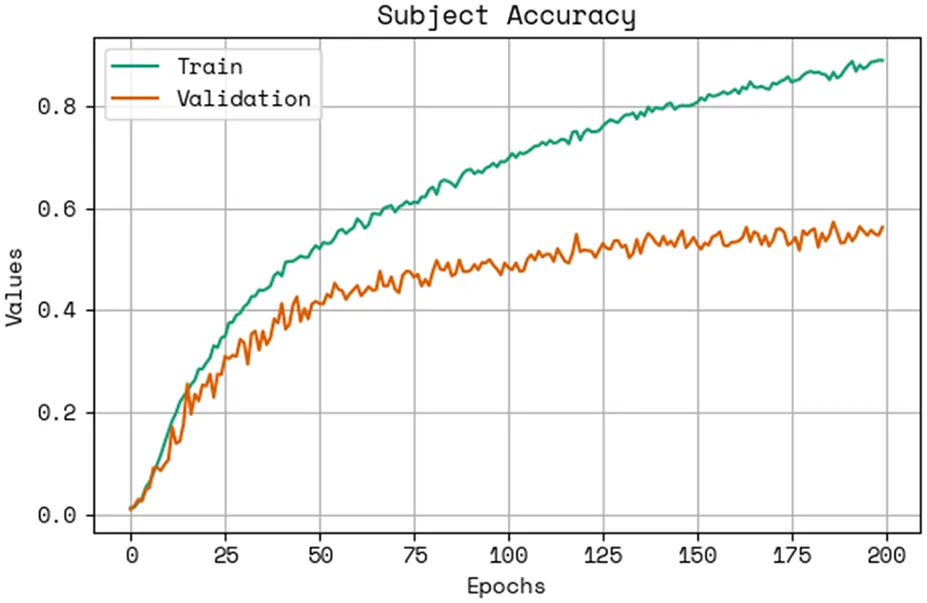
Accuracy curve for person identification under the ECG-ID dataset.

Figure 13 shows the TRAN and VALD accuracy of the FDDR-ID model for person identification under the ECG-ID dataset over 200 epochs. Both curves progressively rise and slowly converge, which shows that the model is learning efficiently. The VALD accuracy constantly remains slightly superior to the TRAN accuracy, implying that the model is not overfitting and generalizes well to unseen data. The fluctuations in accuracy are expected because of the intricacy of the task, but the overall upward tendency highlights the stronger performance and steadiness of the model.

**Figure 14:**
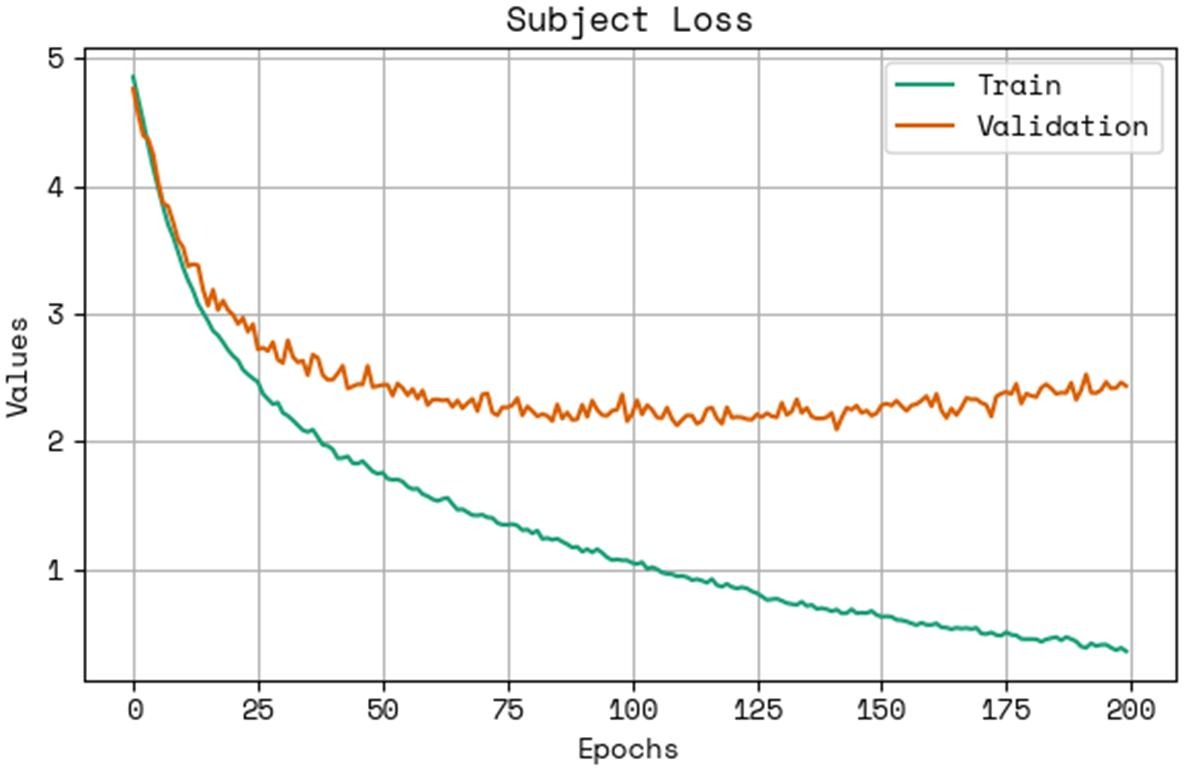
Loss curve for person identification under the ECG-ID dataset.

Figure 14 shows the TRAN and VALD loss of the FDDR-ID technique for person identification under the ECG-ID dataset over 200 epochs. Both curves display a constant downward tendency, demonstrating that the model efficiently minimizes error in learning. The VALD loss remains slightly lower than the training loss through most epochs, implying good generalization and no signs of overfitting. Though some fluctuations are observed, they are becoming gradually constant and steady.

Table 7 shows the training and testing set outcomes of the FDDR-ID technique for age prediction under the ECG-ID dataset. Under the training set, the proposed FDDR-ID model has obtained the following average values: MSE of 8.65549, RMSE of 2.94202, MAE of 1.82438, and R2-Score of 0.94761. Also, under the testing set, the proposed FDDR-ID model has obtained the following average values: MSE of 91.75329, RMSE of 9.57879, MAE of 5.50388, and R2-Score of 0.41572.

**Table 7:** Training and testing sets outcome for age prediction under ECG-ID dataset.

Age Prediction		
Metrics	Training Set	Testing Set
MSE	8.65549	91.75329
RMSE	2.94202	9.57879
MAE	1.82438	5.50388
R2-Score	0.94761	0.41572

**Figure 15:**
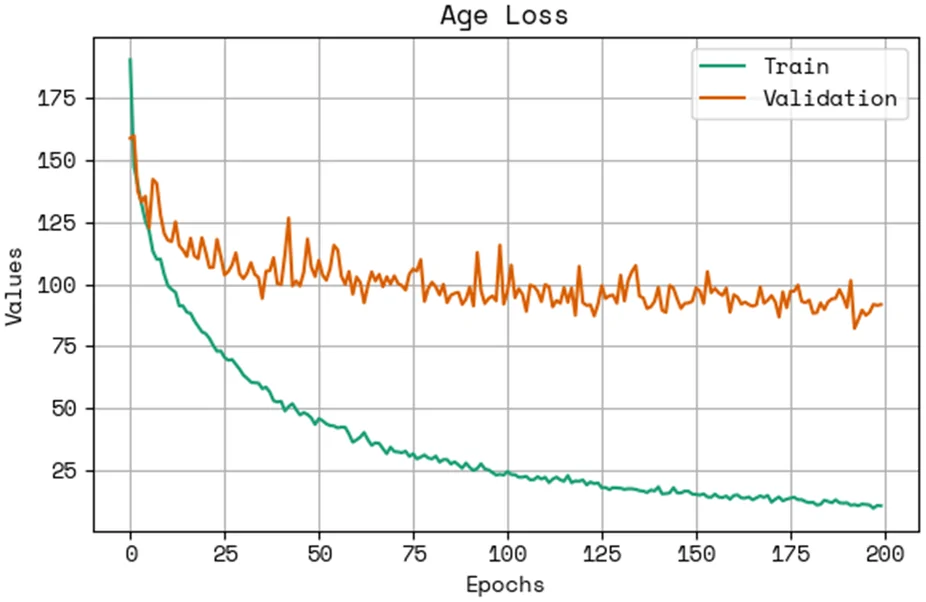
Loss curve for age prediction under the ECG-ID dataset.

Figure 15 shows the TRAN and VALD loss of the FDDR-ID methodology for age prediction under the ECG-ID dataset over 200 epochs. Both curves display a constant downward tendency, signifying that the model effectively minimizes error throughout learning. The VALD loss remains slightly lower than the training loss through most epochs, implying good generalization and no signs of overfitting. However, some fluctuations are observed; they become steadier and more consistent.

Table 8 shows the training and testing set outcomes of the FDDR-ID method for gender identification under the ECG-ID dataset. On the training set, the proposed FDDR-ID technique has attained average $accur_{y}$, $preci_{n}$, $recal_{l},\;F1_{score}$, and MCC values of 94.09%, 94.25%, 94.03%, 94.08%, and 88.28%, respectively. Also, on the test set, the proposed FDDR-ID system reached average $accur_{y}$, $preci_{n}$, $recal_{l},\;F1_{score}$, and MCC values of 77.59%, 77.93%, 77.41%, 77.43%, and 55.33%, respectively.

**Table 8:** Training and testing sets outcome for gender identification under ECG-ID dataset.

Gender Identification		
Metrics	Training Set	Testing Set
Accuracy	94.09	77.59
Precision	94.25	77.93
Recall	94.03	77.41
F1-Score	94.08	77.43
MCC	88.28	55.33

**Figure 16:**
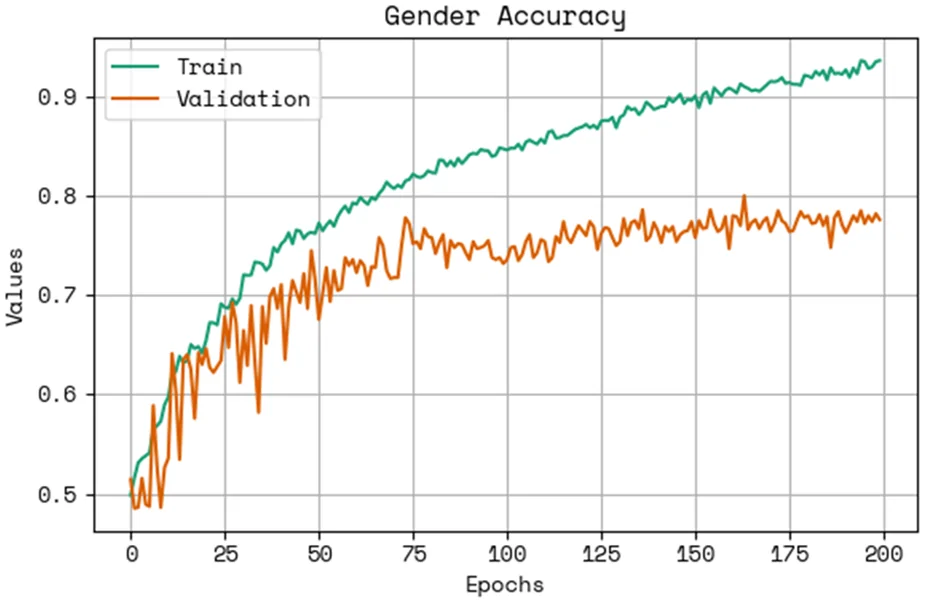
Accuracy curve for gender identification under the ECG-ID dataset.

Figure 16 shows the TRAN and VALD accuracy of the FDDR-ID model for gender identification under the ECG-ID dataset over 200 epochs. Both curves steadily rise and slowly converge, which shows that the model is learning effectively. The VALD accuracy remains somewhat greater than the TRAN accuracy, implying that the model is not overfitting and generalizes well to unseen data. The fluctuations in accuracy are expected owing to the intricacy of the task, but the overall upward tendency indicates stronger performance and model stability.

**Figure 17:**
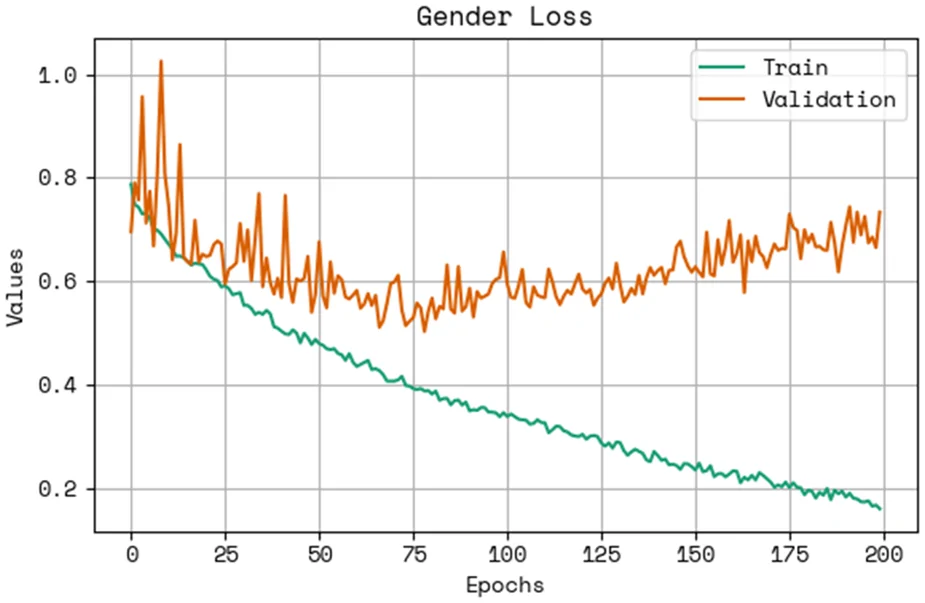
Loss curve for gender identification under the ECG-ID dataset.

Figure 17 shows the TRAN and VALD loss of the FDDR-ID model for gender identification under the ECG-ID dataset over 200 epochs. Both curves reveal a reliable downward tendency, indicating that the model efficiently minimizes error in learning. The VALD loss remains slightly lower than the training loss through most epochs, implying good generalization and no signs of overfitting. Even though some fluctuations are observed, they are becoming gradually constant and steady.

**Figure 18:**
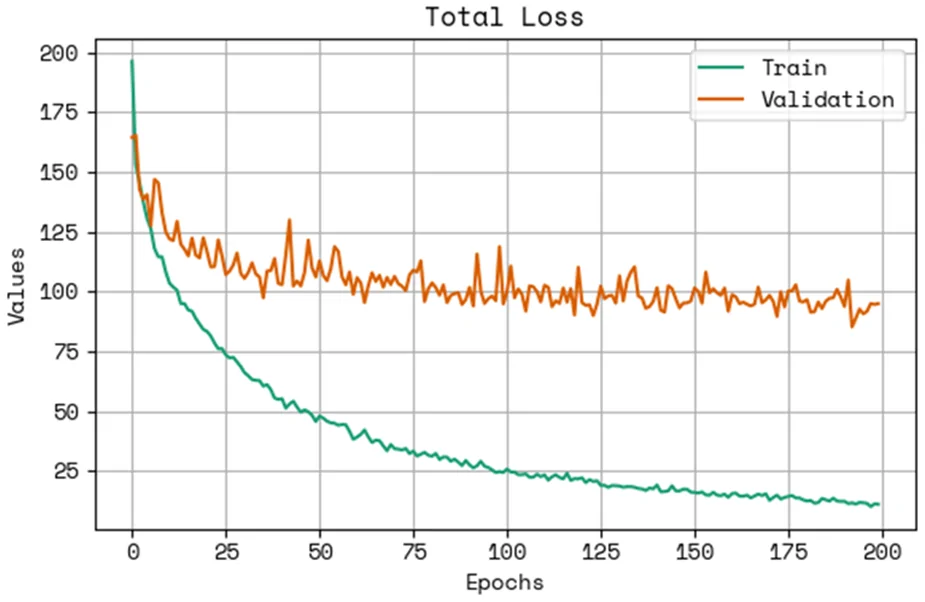
Total loss curve for person identification, age prediction, and gender identification under the ECG-ID dataset.

Figure 18 shows the TRAN and VALD loss for person identification, age prediction, and gender identification under the ECG-ID dataset over a sequence of epochs throughout model training. The green line denotes the training loss, which progressively rises and stabilizes at a lower value, demonstrating that the model is learning efficiently on the training data. The orange line displays the validation loss, which primarily reduces but then fluctuates and stays higher than the training loss. This gap between the two losses implies overfitting, meaning the model performs well on the training data but struggles to generalize to unseen validation data. Overall, while training convergence is obtained, additional models like regularization, early stopping, or dropout can be required to enhance generalization performance.

### 4.3 Comparative analysis

Table 9 shows the comparative analysis of the FDDR-ID method and existing models for person identification (1–37). This study emphasizes that the presented FDDR-ID model has attained greater performance. Based on $accur_{y}$, the proposed FDDR-ID model has gained advanced $accur_{y}$ of 95.01% while the Inception, Squeezenet, ResNet-101, Inception-ResNet-v2, 1D-CNN, SVM, RFT, Googlenet, and DWSC models have acquired lower $accur_{y}$ values of 78.45%, 69.41%, 55.08%, 54.67%, 90.93%, 91.67%, 92.16%, 87.36%, and 88.89%, respectively. Moreover, based on $F1_{Score}$, the presented FDDR-ID system has achieved a better $F1_{Score}\;$ of 94.95%, while the Inception, Squeezenet, ResNet-101, Inception-ResNet-v2, 1D-CNN, SVM, RFT, Googlenet, and DWSC approaches have achieved smaller $F1_{Score}\;$ values of 85.53%, 83.75%, 85.46%, 90.93%, 93.92%, 83.20%, 88.89%, 87.31%, and 88.51%, respectively. The computational time (CT) of dissimilar techniques for the person identification task is also discussed. Minimal CT specifies higher efficiency and earlier processing. The proposed FDDR-ID attains the lowest execution time (3.03 s) compared to existing algorithms, illustrating its computational efficacy and suitability for real-time applications.

**Table 9:** Comparative analysis of FDDR-ID method for person identification.

Person Identification					
Models	$Accur_{y}$	$Preci_{n}$	$Recal_{l}$	$F1_{\mathrm{score}}$	Computational Time (s)
Inception	78.45	89.28	81.32	85.53	4.3
Squeezenet	69.41	90.96	83.52	83.75	4.6
ResNet-101	55.08	85.83	91.23	85.46	5.29
Inception-ResNet-v2	54.67	80.14	82.26	90.93	6.4
1D-CNN	90.93	84.77	94.99	93.92	7.75
SVM Method	91.67	82.69	79.17	83.2	8.23
RFT Model	92.16	87.09	80.23	88.89	5.02
Googlenet	87.36	86.14	88.52	87.31	4.81
DWSC	88.89	89.46	87.58	88.51	3.87
FDDR-ID	95.01	95.33	94.97	94.95	3.03

Table 10 shows the comparative study of the FDDR-ID model and existing systems for age prediction. This study highlights that the presented FDDR-ID system has gained superior performance with the following values: MSE of 5.7910, RMSE of 2.4065, and MAE of 0.4500. Meanwhile, the existing algorithms, such as LSTM, Inception1 D, Transformer, DenseNEt210, MobileNetV2, CORAL, and Randomized Bins, have obtained lower outcomes.

**Table 10:** Comparative analysis of FDDR-ID model for age prediction.

Age Prediction				
Algorithms	MSE	RMSE	MAE	Computational Time (s)
LSTM	9.2540	3.0420	4.6100	7.84
Inception1 D	10.1910	3.1923	5.0900	4.89
Transformer	13.1320	3.6238	5.6900	5.55
DenseNEt210	7.0630	2.6576	5.5500	6.84
MobileNetV2	6.0100	2.4515	4.5800	5.13
CORAL	9.9410	3.1529	5.3900	8.44
Randomized Bins	5.8610	2.4210	4.5500	5.88
FDDR-ID	5.7910	2.4065	0.4500	3.90

Table 11 shows the comparative result analysis of the proposed model with existing models for gender identification. Conventional approaches such as Linear-SVM and K-NN show robust but comparatively moderate outcomes, whereas the Softmax and Segmentation-CNN techniques show good $recal_{l}$ but somewhat lower $preci_{n}$. Overall, the presented FDDR-ID system clearly outperforms other existing models, showing its robustness and effectiveness in accurate Person Identification, age prediction, and gender classification.

**Table 11:** Comparative analysis of FDDR-ID model for gender identification.

Gender Identification					
Models	$Accur_{y}$	$Preci_{n}$	$Recal_{l}$	$F1_{\mathrm{score}}$	Computational Time (s)
Segmentation-CNN	98.90	92.81	91.54	90.35	5.11
LASSO-KNN	99.13	99.03	93.35	93.26	7.82
2D-CNN	99.00	96.74	93.87	90.31	5.41
RNN Algorithm	98.06	95.70	98.34	95.80	3.99
Linear-SVM	97.75	96.61	97.79	95.20	6.37
K-NN Model	97.00	92.95	90.67	91.20	7.78
Softmax	96.82	90.40	99.04	93.68	6.09
FDDR-ID	99.91	99.47	99.47	99.47	3.02

Table 12 shows the computational complexity analysis of dissimilar algorithms over the three datasets—person identification, age prediction, and gender classification—in terms of FLOPs, GPU memory usage, and inference time. Inferior values indicate higher computational efficiency and lesser resource requirements. The proposed FDDR-ID methodology reliably attains minimum FLOPs, reduced GPU memory consumption, and earlier inference time compared to existing techniques for all three tasks (38). These outcomes illustrate that FDDR-ID delivers an effective and lightweight solution while maintaining strong performance, making it suitable for real-time and practical deployment scenarios.

**Table 12:** Computational efficiency for FDDR-ID algorithms.

Methods	FLOPs (G)			GPU (M)			Inference time (ms)		
	Person	Age	Gender	Person	Age	Gender	Person	Age	Gender
SVM	39.90	47.26	48.13	6,337	4,586	6,749	395	219	136
2DCNN-VGGNet	4.90	61.92	29.85	7,930	5,511	3,967	73	434	38
dilated CNN	50.20	45.59	8.20	5,543	2,305	4,269	365	85	183
ResNet 18	1.90	72.54	13.60	7,169	4,267	5,125	95	262	25
Autoencoder	42.20	32.23	51.65	5,150	2,582	2,161	165	185	78
Vanilla CNN	7.20	57.90	13.01	7,012	6,265	7,962	140	329	209
IMPA-CNN	13.23	36.62	76.12	3,059	6,967	3,662	135	447	401
FDDR-ID	0.62	10.66	4.80	932	1,003	845	30	28	14

## 6 Conclusion

This paper presented an FDDR-ID model to achieve robust person identification based on ECG signals under arrhythmic conditions. To achieve this, the proposed FDDR-ID approach primarily pre-processed the ECG signals with three sub-processes: NLM filtering-based denoising, R-peak detection, and segmentation. In addition, to benefit from the efficiency of the DL models, every pre-processed ECG segment is transformed into a 2D representation utilizing STFT to produce spectrograms. For the feature extraction process, a fusion of three DL techniques is employed, namely SqueezeNet, NASNet, and EfficientNet. Furthermore, the SRAE is used for classification with three output branches: person identification, age prediction, and gender classification. The model is trained with adaptive learning by leveraging the Nadam optimizer, guaranteeing faster convergence and enhanced generalization. The performance validation of the proposed FDDR-ID technique is evaluated using the ECG-ID Database and MIT-BIH Arrhythmia Database. An extensive comparison study reported that the presented method outperforms existing state-of-the-art models. The proposed FDDR-ID method also shows important potential for real-world deployment in applications like smart authentication systems, wearable healthcare devices, remote patient monitoring, and secure biometric access platforms. Because of its robust recognition ability under arrhythmic conditions, the technique can support dependable ECG-based authentication and personalized healthcare services. Future combination with IoT-enabled and wearable systems may further improve its practical applicability and societal impact.

Although the proposed methodology showed strong recognition performance under arrhythmic conditions, multiple limitations remain. The recent algorithm relies primarily on STFT-based spectral depictions and a predefined deep feature fusion strategy, which could not completely capture all nonlinear and unclear characteristics present in extremely noisy or pathological ECG signals. Furthermore, the classification methodology could display sensitivity to severe signal variations produced by subject variability, acquisition artifacts, and complex cardiac abnormalities. The proposed technique was also evaluated on a limited number of benchmark databases, which may disturb generalization over larger real-world scenarios.

Future work should focus on improving robustness and generalization under noisy and pathological ECG disorders through the addition of progressive similarity and classification approaches. In particular, fuzzy similarity-based methodologies can be applied to handle improbability and unclear ECG patterns. Besides, more sophisticated classification architectures, with graph-based learning, attention-based models, and adaptive ensemble algorithms, additionally enhance discriminative ability and strength. We will focus on addressing potential data imbalance issues that may disturb method generalization and classification outcomes. Approaches like the Synthetic Minority Over-sampling Technique (SMOTE) will be explored to produce balanced training instances and enhance representation of underrepresented class labels. Future studies will also examine learned signal illustrations and evaluate the framework on larger and more varied ECG databases collected under real-world conditions.

## Data Availability

The datasets presented in this study can be found in online repositories. The names of the repository/repositories and accession number(s) can be found at https://physionet.org/content/ecgiddb/1.0.0/. The dataset also can also be retrieved from the Kaggle dataset platform at https://www.kaggle.com/datasets/bjoernjostein/ecgid-database. The names of the repository/repositories and accession number(s) can be found at https://physionet.org/content/ecgiddb/1.0.0/. The dataset also can also be retrieved from the Kaggle dataset platform at https://www.kaggle.com/datasets/bjoernjostein/ecgid-database.
